# Characterization of fungal pathogens and germplasm screening for disease resistance in the main production area of the common bean in Argentina

**DOI:** 10.3389/fpls.2022.986247

**Published:** 2022-09-07

**Authors:** Gisel Taboada, Carla L. Abán, Guadalupe Mercado Cárdenas, Yamila Spedaletti, Mónica Aparicio González, Efrain Maita, Pablo Ortega-Baes, Marta Galván

**Affiliations:** ^1^Consejo Nacional de Investigaciones Científicas y Técnicas (CONICET) CCT-Salta, Salta, Argentina; ^2^Instituto Nacional de Tecnología Agropecuaria (INTA) EEA Salta, Salta, Argentina; ^3^Laboratorio de Investigaciones Botánicas (LABIBO), Facultad de Ciencias Naturales, Universidad Nacional de Salta, Salta, Argentina

**Keywords:** white mold, angular leaf spot, web blight, Rhizoctonia root rot, *Sclerotinea sclerotiorum*, *Pseudocercospora griseola*, *Rhizoctonia solani*, fungal diseases

## Abstract

The common bean (*Phaseolus vulgaris* L.) is the most important grain legume in the human diet, mainly in Africa and Latin America. Argentina is one of the five major producers of the common bean in the world, and the main cultivation areas are concentrated in the northwestern provinces of this country. Crop production of the common bean is often affected by biotic factors like some endemic fungal diseases, which exert a major economic impact on the region. The most important fungal diseases affecting the common bean in Argentina are white mold caused by *Sclerotinia sclerotiorum*, angular leaf spot caused by *Pseudocercospora griseola*, web blight and root rot caused by *Rhizoctonia solani*, which can cause production losses of up to 100% in the region. At the present, the most effective strategy for controlling these diseases is the use of genetic resistance. In this sense, population study and characterization of fungal pathogens are essential for developing cultivars with durable resistance. In this review we report diversity studies carried out on these three fungal pathogens affecting the common bean in northwestern Argentina, analyzing more than 200 isolates by means of molecular, morphological and pathogenic approaches. Also, the screening of physiological resistance in several common bean commercial lines and wild native germplasm is reviewed. This review contributes to the development of sustainable management strategies and cultural practices in bean production aimed to minimize yield losses due to fungal diseases in the common bean.

## Introduction

The American continent is the center of domestication of many crops that are essential in the diet of human populations, such as maize (*Zea mays* L.), tomato (*Solanum tuberosum* L.), potato (*Solanum lycopersicum* L.), and common bean (*Phaseolus vulgaris* L.). The common bean is the dry grain legume most consumed in the world due to its high content of proteins, carbohydrates, fibers and minerals, being a main part of the diet of many countries in America and Africa (Broughton et al., 2003; Gepts et al., 2008). Domestication of the common bean occurred independently in two regions throughout the continent. Therefore two major gene pools, named Mesoamerican and Andean, are recognized in the population structure of the wild and the domesticated beans (Papa and Gepts, 2003; Papa et al., 2005, 2007; Rossi et al., 2009; Cortinovis et al., 2020; Tobar Piñón et al., 2021). Domesticated beans further diverged into genetically distinct races giving rise to the diversity of market types known today (Kwak and Gepts, 2009; Tobar Piñón et al., 2021).

Dry beans world production reached 27.5 million tons in 2020 (FAO, 2022). Argentina is among the top five common bean exporting countries and exports 90% of its production, supplying the crop to many Latin American countries (FAO, 2022). Bean production is located in the northwestern region of Argentina (NWA), comprising the provinces of Jujuy, Salta, Tucumán, Santiago del Estero and Catamarca. These regions are characterized by a great climatic and environmental heterogeneity, reaching a common bean production of 633.823 tons per year (FAO, 2022). Within this heterogeneous landscape, biotic stress is one of the main limiting factors for bean production (Basavaraja et al., 2020).

The common bean is affected by numerous diseases caused by fungi, viruses, bacteria and nematodes that affect production in different ways. To date, more than 200 diseases that cause significant losses in bean yield have been reported (Schwartz and Pastor Corrales, 1989; Assefa et al., 2019). Although NWA presents adequate conditions for common bean development, its production is constrained by different phytosanitary problems and the lack of disease resistance varieties. The main fungal diseases that affect bean production in the region are white mold [*Sclerotinia sclerotiorum* (Lib.) de Bary], angular leaf spot [*Pseudocercospora griseola* (Sacc.) Crous and U. Braun], web blight and Rhizoctonia root rot (*Rhizoctonia solani* Kühn). These are the most dispersed diseases in the different bean production areas in the country and are the most important due to the economic losses they cause (Vizgarra et al., 2011, 2012).

At the present, the most effective strategy for controlling these diseases is the use of genetic resistance. In this sense, population study and characterization of fungal pathogens are essential for developing cultivars with durable resistance. In this review we report diversity studies carried out on these three fungal pathogens affecting common bean in northwestern Argentina, analyzing more than 200 isolates by means of molecular, morphological and pathogenic approaches. Also, the screening of physiological resistance in several common bean commercial lines and wild native germplasm are covered in this review.

## White mold

**FIGURE 1:**
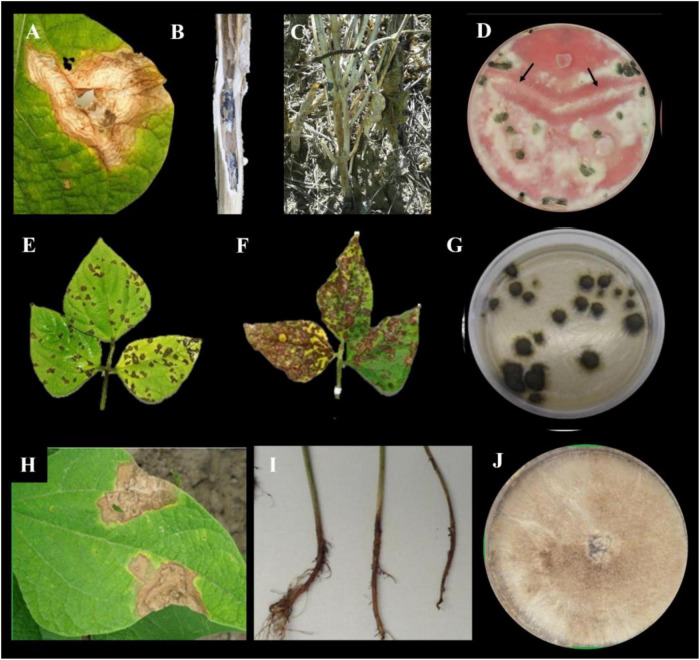
White mold symptoms on common bean **(A)** leaf and **(B)** stem. **(C)** Common bean cultivar showing white mold symptoms. **(D)** Mycelial compatibility test between three isolates of *Sclerotinia sclerotiorum*. The arrows indicate incompatible reactions with band of aerial mycelium in the interaction zone. **(E,F)** Common bean cultivars showing a susceptible reaction to *Pseudocercospora griseola*. **(G)** Andean *P. griseola* isolate. **(H)** Web blight symptoms on common bean leaf. **(I)** Rhizoctonia root rot symptoms on common bean. **(J)** *Rhizoctonia solani* isolates obtained from common bean seed and soil.

White mold (WM) caused by *Sclerotinia sclerotiorum* is one of the most destructive fungal diseases of the common bean worldwide (Boland and Hall, 1994). This necrotrophic fungus has a broad host range of more than 400 species in 75 plant families, including field crops, cereals, horticultural crops, trees, shrubs and several weed plants (Boland and Hall, 1994). Some of the major economic crops affected include dry bean, potato, soybean [*Glycine max* (L.) Merr.], sunflower (*Helianthus annuus* L.), canola (*Brassica napus* L.), lettuce (*Lactuca sativa* L.), carrot (*Daucus carota* L.), and pea (*Pisum sativum* L.) (Carpenter et al., 1999; Mert-Türk et al., 2007; Hemmati et al., 2009; Attanayake et al., 2013; Lehner et al., 2015; Abán et al., 2018; Panullo et al., 2018). In Argentina, WM has been detected in all bean production areas, reaching seed yield and quality losses up to 80–100% on susceptible common bean cultivars under favorable weather conditions (Singh and Schwartz, 2010). WM disease affects all aerial parts of plants regardless of the growth stages of the plant. Disease symptoms of WM typically begin with water-soaked lesions on leaves and stems (Figure 1). As the disease progresses, a thick white mycelium growth followed by hard black sclerotia is observed in internal and external tissues of the plant, which causes distal portions of the plant to wilt and then become necrotic (Steadman and Boland, 2005). Eventually, the plant will appear bleached in color, with plant parts showing shredded characteristics due to tissue breakdown (Purdy, 1979). Sclerotia can germinate myceliogenically to infect adjacent plant tissues and carpogenically via apothecia from which ascospores are dispersed within the crop. Sclerotia eventually fall to the ground as infected stems dry out and the host plant dies. These sclerotia serve as the primary source of inoculum of the disease (Bolton et al., 2006). The longevity of sclerotia in the soil varies from 1 year (Brustolin et al., 2016) to up to 8 years (Adams, 1979), making this pathogen extremely hard to control in the field. WM disease can also be spread by the movement of seeds contaminated and sclerotia mixed with seeds from one field to another, irrigation runoff water and wind-blown ascospores, which can travel a considerable distance of 3–4 km between fields (Cubeta et al., 1997; Steadman and Boland, 2005).

*Sclerotinia sclerotiorum* is a homothallic and haploid fungus that can reproduce asexually (clonally) by means of mycelium or sexually by means of self-fertilization or recombination (Attanayake et al., 2014) to produce apothecia with ascospores. However, sexual reproduction in haploid fungi is frequently equivalent to clonal reproduction (Billiard et al., 2012) because the genetic exchange that exists is scarce and is not enough to break the predominant pattern of clonal population structure (Tibayrenc and Ayala, 2012). According to recent research, mycelial compatibility groups (MCGs) are useful as a rough measure of standing genotypic diversity but are not adequate to infer population genetic processes (Kamvar and Everhart, 2019; Figure 1). However, other studies have suggested taking into account the structure imposed by the MCGs in addition to a set of molecular markers in population analyses (Lehner and Mizubuti, 2017; Lehner et al., 2019; Silva et al., 2021). Over the past few years, the population structure of *S. sclerotiorum* has been extensively documented from different host crops and from different regions in the world (Atallah et al., 2004; Sexton et al., 2006; Hemmati et al., 2009; Ekins et al., 2011; Attanayake et al., 2013; Clarkson et al., 2013, 2017; Aldrich-Wolfe et al., 2015; Dunn et al., 2017; Panullo et al., 2018; Faraghati et al., 2022). In early studies, *S. sclerotiorum* populations exhibited a predominantly clonal population structure with low genetic diversity based on MCGs and DNA fingerprinting genotypes (Kohli et al., 1992; Cubeta et al., 1997; Hambleton et al., 2002). However, in subsequent studies, evidence of recombinant populations and mixed population structures with high rates of genetic variability have been reported using microsatellite (SSR) markers and linkage disequilibrium measures (Atallah et al., 2004; Sexton and Howlett, 2004; Hemmati et al., 2009; Attanayake et al., 2014; Panullo et al., 2018). Whether a pathogen population is clonal or recombining is best addressed by studying the association of alleles among different loci through the index of association (*I*_*A*_) (Smith et al., 1993; Milgroom, 1996) based on clone-corrected data to reduce the bias produced by overrepresented data and to increase sensitivity in the detection of recombination (Milgroom, 1996). Recombinant populations with high rates of genetic variability tend to have a high evolutionary potential and therefore are more likely to overcome host resistance. Thus, the presence of *S. sclerotiorum* recombinant populations in a particular region is of great interest to the delineation of strategies for WM management and crucial for breeders seeking to develop new resistant cultivars (Milgroom, 1996; McDonald and Linde, 2002).

Despite the fact that the bean crop is cultivated in many countries, the genetic diversity and population structure of *S. sclerotiorum* in common bean crops have only been analyzed in Brazil (Lehner et al., 2015, 2017, 2019; Silva et al., 2021), the United States (Kamvar et al., 2017) and Argentina (Abán et al., 2018, 2021). In Brazil, the first study using microsatellite markers analyzed 79 isolates and reported high genotypic variability among *S. sclerotiorum* isolates (Gomes et al., 2011). However, in a subsequent study using linkage disequilibrium measures, Lehner et al. (2015) reported that despite the relatively high genotypic diversity observed among isolates, the SSR loci were in linkage disequilibrium, and thus, the *S. sclerotiorum* population had a clonal genetic structure. These results were later supported in larger studies, where the pathogen population of Brazil not only remained clonal but also structured according to MCGs (Lehner et al., 2019; Silva et al., 2021). Silva et al. (2021) analyzed 238 isolates, and only 22 MCGs and 64 SSR haplotypes were found, with no association between SSR haplotypes and MCGs. Although their clonal lineages were widely distributed in space and persistent over time, evidence of some degree of outcrossing was detected (Silva et al., 2021). In the case of common bean fields in the United States, Kamvar et al. (2017) reported that *S. sclerotiorum* populations had a clonal population structure with low genetic diversity using MCGs and SSRs. In this study, 366 isolates were analyzed from production fields and WM screening nurseries from dry bean cultivars among different geographic locations in the United States (320), France (22), Mexico (18), and Australia (6). A total of 165 MLH and 87 MCGs were observed, with no relationship between SSR haplotypes and MCGs. In contrast to Brazil, the United States populations from dry bean fields were structured by region, and no evidence of structuring by MCGs was detected.

In Argentina, the molecular and morphological identification of 116 *S. sclerotiorum* isolates from the main common bean production area was reported by Abán et al. (2018). Morphological identification was confirmed by PCR amplification and sequencing of the rRNA ITS region, which presented 100% similarity compared to *S. sclerotiorum* sequences. In addition, a first approach of the mode of reproduction and population structure was analyzed by means of MCGs and URPs (Universal Rice Primers) molecular haplotypes (Abán et al., 2018). A total of 52 MCGs and 59 URP haplotypes were found. All the MCGs were location specific, while only 12% of the URP haplotypes were shared among locations. Moreover, most of the isolates were highly aggressive, while no variation among locations was observed. Based on measures of multilocus linkage disequilibrium, the occurrence of both clonal and sexual reproduction was suggested in *S. sclerotiorum* populations from common bean fields in northwestern Argentina (Abán et al., 2018). Since most population structure analyses are based on SSR markers, a later study based on microsatellite markers was performed (Abán et al., 2021). In this study, 109 isolates of *S. sclerotiorum* from six dry bean fields in the main production area of Argentina were analyzed using nine microsatellite loci. A total of 30 SSR haplotypes were identified, of which 18 haplotypes were unique. Population genetic structure analysis based on linkage disequilibrium analysis suggested the occurrence of both modes of reproductive behavior, with sexual recombination being the most frequent (Abán et al., 2021). The high levels of recombination and gene flow detected in this study highlighted the need for breeding programs to develop new cultivars resistant to WM.

The integrated management of the disease includes the use of resistant or tolerant cultivars, cultural practices, fungicide applications during the flowering stage, upright growth habit plants, wide row spacing in combination with low plant density (Vieira et al., 2012, 2022), and biological control by different antagonistic fungi, bacteria and organic amendments, which has been recently reviewed by Smolińska and Kowalska (2018). Regarding biological control, different native strains of the genus *Bacillus* with the potential to control WM on bean seeds and seedlings in NWA, was reported by Sabaté et al. (2018). To date, however, there are no known common bean cultivars with complete resistance and current biological control methods are rarely sufficient to completely reduce the population of the pathogen; thus, fungicide applications remain the most effective tool for disease control, but overuse and misuse of fungicides increase the risk of fungicide resistance emergence (McDonald and Linde, 2002). Moreover, populations with frequent outcrossing will have relatively higher levels of genetic diversity; thus, the risk of fungicide resistance emergence is increased (McDonald and Linde, 2002). Hence, the best strategy to minimize yield losses and reduce production costs in a sustainable farming context is the use of varieties with genetic resistance to WM. When evaluating genetic resistance to WM, physiological resistance and disease avoidance traits are considered for the selection of resistant genotypes. Both characteristics are quantitatively inherited, and resistance and avoidance QTLs have already been identified (Mkwaila et al., 2011; Pérez-Vega et al., 2012; Miklas et al., 2013; Vasconcellos et al., 2017). A comparative map including 27 QTLs for WM resistance and 36 QTLs for disease-avoidance traits was developed by Miklas et al. (2013). Vasconcellos et al. (2017) identified 37 QTLs located in 17 loci, nine of which were defined as meta-QTLs. These are robust consensus QTLs representing effects across different environments, genetic backgrounds and related traits. Moreover, within the confidence interval for five of the meta-QTLs, candidate genes expressed under *S. sclerotiorum* infection, such as ethylene-responsive transcription factor, peroxidase, cell wall receptor kinase *COI1* and MYB transcription factor were found. These nine meta-QTLs are recommended as potential targets for molecular marker-assisted selection for partial resistance to WM in the common bean (Vasconcellos et al., 2017).

Currently, there are no commercial bean varieties available with WM resistance. In previous studies, however, low levels of resistance have been reported in genotypes of Mesoamerican origin (Ender and Kelly, 2005; Pascual et al., 2010; Mkwaila et al., 2011) and in wild beans (Terpstra and Kelly, 2008; Mkwaila et al., 2011), and high levels of resistance have been reported in genotypes of Andean origin (Maxwell et al., 2007; Singh et al., 2007; Pascual et al., 2010; Mkwaila et al., 2011; Soule et al., 2011; Pérez-Vega et al., 2012). In addition, higher levels of WM resistance have been introgressed from interspecific crosses with secondary gene pool *Phaseolus* species such as *P. coccineus*, *P. polyanthus*, and *P. costaricensis* (Schwartz et al., 2006; Singh et al., 2009, 2013, 2014).

In Argentina, the physiological resistance of 20 common bean accessions (cultivars and lines) was assessed at 7, 14, and 21 days post-inoculation with five genetically distinct isolates of *S. sclerotiorum* collected from the main common bean growing area of NWA (Aban et al., 2020). These isolates were previously characterized using URP and SSR molecular markers, MCGs and pathogenicity tests (Abán et al., 2018, Aban et al., 2020). Based on the modified Petzoldt and Dickson scale (Terán et al., 2006), all cultivars and lines were susceptible at the end of the assessment, except line A 195, which was resistant to WM against the five isolates tested and was significantly different from all accessions. Line A 195 is a registered WM-resistant germplasm (Singh et al., 2007) from the Centro Internacional de Agricultura Tropical (CIAT) in Colombia. In previous studies, line A 195 showed partial levels of resistance to different highly and weakly aggressive *S. sclerotiorum* isolates (Viteri et al., 2015), including one pathogen isolate (ARS12D) collected in Salta, Argentina in 2012 (Viteri et al., 2015). Regional common bean breeding programs aimed at obtaining broadly adapted cultivars with durable resistance to WM should account for the regional variation within a pathogen population to ensure the development and release of durable WM-resistant common bean cultivars. Line A 195 is a promising parental genotype to be used in regional breeding programs.

## Angular leaf spot

Angular leaf spot (ALS), caused by the ascomycota fungus *Pseudocercospora griseola*, is one of the diseases that causes great economic losses to bean production (Schoch et al., 2009). This pathogen is an important etiological agent mainly in countries with subtropical and tropical climates, such as Brazil, Argentina, Bolivia and African countries (Guzmán et al., 1995; Pastor-Corrales et al., 1998; Vizgarra et al., 1999, 2011; Ploper et al., 2002, 2016; Espeche et al., 2018). In recent years, the incidence of the disease has increased, causing great economic losses, favored by the monoculture system and the narrow genetic base of the commercial bean varieties. In Argentina, yield losses in common bean crops range from 20 to 50% (Stenglein, 2007), and in other regions, such as Brazil and African countries, yield losses can reach up to 80% of the total crop production (De Jesus et al., 2007; Singh and Schwartz, 2010).

ALS disease is mainly destructive in warm and humid areas, affecting the yield and quality of bean seeds. Symptoms are visible on leaves and pods, which present angular brown interveinal spots and circular brown lesions, respectively (Figure 1). The spots on the leaves eventually coalesce, causing premature defoliation (Crous et al., 2006). The pathogen conidia are spread mainly by wind and water droplets. However, agricultural practices have a great influence on the spread of the disease, being carried by agricultural implements and contaminated seeds that facilitate pathogen transmission.

**FIGURE 2:**
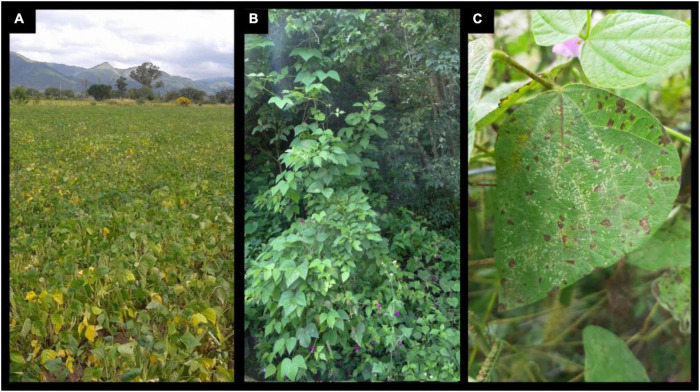
**(A)** Plants showing angular leaf spot symptoms in a common bean field in northwestern Argentina. **(B)** Argentinean wild bean exhibiting its characteristic indeterminate growth habit. **(C)** Wild bean showing angular leaf spot symptoms.

In Argentina, ALS is considered one of the most destructive and problematic diseases for bean production (Vizgarra et al., 2011, 2012, 2016; Espeche et al., 2018). In NWA ALS is a widely distributed fungal disease, particularly in the south of Salta and southeast of Catamarca, mainly in black bean cultivars and in seasons with above-average rainfall during the reproductive period of the crop (Ploper et al., 2016). Under high disease pressure, a substantial reduction in leaf area is observed and the photosynthetic capacity of bean plants decreases during grain filling, when the demand for photosynthates is the highest (Figure 2; Cole, 1966; Hagedorn and Wade, 1974; Schwartz and Galvez, 1980; Cardona Mejía et al., 1995).

Knowledge of the genetic variability of the pathogen population present in each crop-producing region is extremely important for the development of effective management strategies. The ALS pathogen is known for the wide virulence diversity exhibited by isolates from different locations. *P. griseola* pathotypes are defined based on the pathogenicity reaction to a set of 12 common bean differential genotypes (Pastor-Corrales and Jara, 1995; Supplementary Table 1). Based on their reaction to ALS differential cultivars, all *P. griseola* pathotypes (known as races) are separated into Andean and Mesoamerican pathotype groups that correspond to the two common bean gene pools, sustaining the coevolution of the pathogen with its common bean host (Guzmán et al., 1995; Mahuku et al., 2002a; Stenglein and Balatti, 2006; Rezene et al., 2018). Isolates obtained from Andean cultivars were virulent only in Andean bean differential cultivars, which is why these races were called Andean, while isolates from Mesoamerican cultivars were virulent in Mesoamerican ones (Beebe and Pastor-Corrales, 1991; Mahuku et al., 2002a; Stenglein and Balatti, 2006). The existence of a third group of races, named Afro-Andean group, capable of infecting both Andean and Mesoamerican differential cultivars has been reported (Mahuku et al., 2002a,b; Wagara et al., 2004, 2011; Serrato-Diaz et al., 2020). The set of differential cultivars has been widely used throughout the world, allowing the comparison of *P. griseola* races between different localities, countries and even continents. The isolation and characterization of *P. griseola* in Argentina was first reported by Stenglein and Balatti (2006). In this study, 45 isolates collected within the main common bean production area in NWA were classified into 13 races based on the set of bean differential cultivars. Some races, such as 63–15 and 63–7, occurred more frequently than others with the coexistence of different races in certain areas of production (Stenglein and Balatti, 2006). The most pathogenic race was 63–63 reported in Zárate, Tucumán. Races that overcome the resistance of all differential cultivars have been reported in Argentina, Central America, Brazil, and Africa, suggesting the need to expand the number of differential cultivars to better identify these pathotypes (Stenglein and Balatti, 2006; Nay et al., 2019b). In this sense, new genotypes have been proposed as candidates to expand the standard set of differential cultivars (Nay et al., 2019b).

DNA sequence-based comparisons are of great importance to determine the diversity of a pathogen in a region and ensure the availability of an up-to-date barcode that provides meaningful information for plant health (Crous et al., 2013). The ITS region has been widely used by mycologists as a standard barcode, and ITS sequences are currently available for several fungal species identified in public databases (Begerow et al., 2010; Schoch et al., 2012; Rezaee Danesh and Demir, 2020). With respect to *P. griseola*, Aparicio (2020) reported the taxonomical identification of Argentinian pathotypes based on ITS sequences, differentiating the isolates of *P. griseola* f. *mesoamericana* from the isolates of *P. griseola* f. *griseola*, generating a phylogenetic tree similar to that previously obtained by Crous et al. (2006). In addition, polymorphic sites in the sequences of the ITS 1 and ITS 2 regions were identified, which are useful for the development of diagnostic specific oligonucleotides based on the single nucleotide polymorphisms (SNPs) detected.

Several molecular markers have been used to analyze ALS pathogen variability (Guzmán et al., 1999; Mahuku et al., 2002b,^2009^; Stenglein and Balatti, 2006; Abadio et al., 2012; Ddamulira et al., 2014; Nay et al., 2019a). However, finding genetically accurate and operationally simple markers for the study of *P. griseola* variability is not an easy task (Mahuku et al., 2002b). In Argentina, high levels of genetic diversity were observed within the Mesoamerican and Andean groups of the fungus using dominant molecular markers (Stenglein and Balatti, 2006; Aparicio, 2020), in agreement with previous reports from Africa and Brazil (Mahuku et al., 2002b; Abadio et al., 2012). Molecular analyses of Argentinean *P. griseola* isolates performed with RAPD and ISSR markers (Stenglein, 2007) significantly distinguished between the Mesoamerican and Andean isolates; however, unique band patterns or haplotypes were generated for less than 50% of the isolates analyzed. Guzmán et al. (1999) developed specific primers to identify *P. griseola* isolates of each gene pool. However, these specific primers were only efficient in differentiating isolates of Andean origin when used on Argentinean isolates (Aparicio, 2020), demonstrating the wide variability exhibited by isolates from different regions. On the other hand, URP markers were found to be useful tools to differentiate ALS pathogen isolates, being even more efficient than RAPD and ISSR markers (Aparicio, 2020).

Diversity studies of isolates from Argentina showed that *P. griseola* had great pathogenic variability (Stenglein, 2007; Aparicio, 2020). Although Mesoamerican isolates of *P. griseola* had greater genetic diversity than the Andean isolates (Wagara et al., 2004), Aparicio (2020) reported a greater diversity in the Andean group. This may be due to the introgression of genes from the Mesoamerican to Andean isolates, which was also suggested by Stenglein (2007), since in this region, both types of beans (Mesoamerican and Andean) are grown. Moreover, in the same leaf of a bean plant, isolates belonging to the Mesoamerican and Andean groups can be found (Guzmán et al., 1999; Stenglein and Balatti, 2006; Stenglein, 2007; Crous et al., 2013). Based on what is known about the coevolution between the gene pools of the host and the pathogen of the common bean and the high virulence and potential for overcoming resistance of the pathogen, Andean and Mesoamerican resistance gene pyramiding would be the most appropriate strategy to generate cultivars with durable ALS resistance (de Carvalho et al., 1998; Sartorato et al., 1999; Corrêa et al., 2001; Namayanja et al., 2006; Gonçalves-Vidigal et al., 2011, 2013; Vizgarra et al., 2011; Oblessuc et al., 2012, 2013, 2015; Goncalves-Vidigal et al., 2020).

To date, integrated management is the most widely used strategy for ALS management, which involves cultural methods (crop rotation, seed sanitation and adequate planting dates), chemical methods (fungicide use) and biological methods (resistant genotypes). Numerous studies agree that the most sustainable strategy to control ALS disease is the use of resistant cultivars. Many genotypes were evaluated in search of new sources of ALS resistance, including the identification of SNP markers to be used in breeding assisted selection and pyramidization of resistance genes (Singh and Schwartz, 2010; Nay et al., 2019b). The TUC 550 cultivar, was the first black bean cultivar with resistance to ALS in Argentina developed by the Estación Experimental Agropecuaria Obispo Colombres (EEAOC) from germplasm introduced from CIAT. This cultivar was released in 2010 and showed resistance to different races of the pathogen that were the most prevalent in bean cultivated areas (Vizgarra et al., 2018). These results highlight the importance of knowing the local variability of *P. griseola* isolates to generate genotypes adapted to the region and with durable resistance over time. Other cultivars, such as MAB 333 and MAB 336, introduced from CIAT reported high levels of resistance to angular leaf spot in field evaluations (Vizgarra et al., 2011). Recently, the TUC180 and TUC241 cultivars, that are red and cranberry type beans, were reported to be resistant to races 63–7 and 31–0 by Aparicio (2020). These genotypes are new potential parents for future combinations, considering that breeding for ALS resistance should be continuous because of the high pathogenic variability exhibited by the pathogen.

The identification of new resistance genes is a major goal for geneticists to broaden the common bean genetic base against the ALS pathogen, to understand the nature of defense genes and to define haplotypes for marker design to assist in breeding. Resistance to the ALS pathogen is largely conferred by single dominant resistance genes, named *Phg-1*, *Phg-2*, and *Phg-3*, but a quantitative nature of resistance that includes two major QTLs named *Phg-4* and *Phg-5* has also been reported (de Carvalho et al., 1998; Sartorato et al., 1999; Corrêa et al., 2001; Teixeira et al., 2005; Namayanja et al., 2006; Chataika et al., 2010; Gonçalves-Vidigal et al., 2011, 2013; Oblessuc et al., 2013, 2012; Keller et al., 2015; Nay et al., 2018, 2019a). The *Phg-1*, *Phg-4*, and *Phg-5* loci are from common bean cultivars of the Andean gene pool, whereas *Phg-2* and *Phg-3* are from beans of the Mesoamerican gene pool. The *Phg-1* locus mapped on chromosome Pv01 in the AND 277 cultivar (Gonçalves-Vidigal et al., 2011), the *Phg-2* locus mapped on chromosome Pv08 in México 54 cultivar and its allele *Phg-2^2^* is present in BAT 332 (Sartorato et al., 1999; Namayanja et al., 2006), and the *Phg-3* locus mapped on Pv04 in the Ouro Negro cultivar (Corrêa et al., 2001; Faleiro et al., 2003; Gonçalves-Vidigal et al., 2013). On the other hand, the major QTL *Phg-4* mapped on chromosome Pv04 in the G5686 and CAL 143 cultivars (Mahuku et al., 2009; Oblessuc et al., 2012; Keller et al., 2015; Souza et al., 2016), and the QTL *Phg-5* mapped on Pv10 in the CAL 143 and G5686 cultivars (Oblessuc et al., 2012, 2013; Keller et al., 2015; Souza et al., 2016).

Currently, breeding is based on a few well-characterized single resistance genes that are easily transferred to elite commercial cultivars (Nay et al., 2019b). However, due to the wide virulence diversity of *P. griseola*, there is a high risk of losing this resistance. Therefore, new breeding strategies based on a broad diversity of qualitative and quantitative spectra of resistance genes are essential for the development of cultivars with durable resistance (Nay et al., 2019b).

Until a few years ago, most ALS resistance studies were based on biparental mapping populations with the identification of associated markers that were often polymorphic only in segregating populations from specific crosses. Currently, with the availability of a reference genome of common bean (Schmutz et al., 2014; Vlasova et al., 2016) and the development of high-throughput genotyping platforms (Hyten et al., 2010; Goretti et al., 2014; Gujaria-Verma et al., 2016; Raatz et al., 2019), genome-wide association studies (GWAS) have become an efficient and powerful tool for the discovery of novel ALS resistance genes (Perseguini et al., 2016; Zuiderveen et al., 2016; Tock et al., 2017; Fritsche-Neto et al., 2019; Nay et al., 2019a; Vidigal Filho et al., 2020). Perseguini et al. (2016), using GWAS with 180 common bean accessions, identified QTLs controlling resistance to anthracnose and ALS diseases. A total of 11 SSRs and 17 SNPs associated with resistance to race 0–39 of *P. griseola* were detected. The authors reported three SNP markers, two located on chromosome Pv03 and one on Pv07, that were associated with both diseases. Nay et al. (2019a) conducted GWAS in a large common bean panel, which included the ALS most resistant genotypes available at CIAT, and tested it under greenhouse and field conditions at multiple sites in Colombia and Uganda. A major ALS resistance locus conferring resistance in all trials was detected on chromosome Pv08, coinciding with the previously characterized resistance locus *Phg-2* (Sartorato et al., 1999). The resistance locus *Phg-4* on chromosome Pv04 was effective against one particular pathotype. Moreover, DNA sequence-based clustering identified eleven functional haplotypes at *Phg-2*; one conferred broad-spectrum ALS resistance, and six showed pathotype-specific effects (Nay et al., 2019a). The authors highlighted the importance of ALS pathotype specificity for durable resistance management strategies in common bean. Fritsche-Neto et al. (2019) performed GWAS in 60 inbred elite lines from Brazil and evaluated them under field conditions, identifying one SNP associated with ALS resistance loci on chromosome Pv10 and two SNPs associated with anthracnose resistance loci on chromosome Pv02. Vidigal Filho et al. (2020) conducted a GWAS approach using 115 Brazilian accessions and reported SNP markers associated with resistance to race 31–23 of *P. griseola*, which mapped on chromosomes Pv02 and Pv04, whereas for race 63–39, SNPs were mapped on chromosomes Pv03, Pv06, and Pv08. Recently, de Almeida et al. (2021) performed GWAS and linkage mapping approaches to identify ALS resistance loci at different plant growth stages. Different QTLs were detected showing a different quantitative profile of the disease at different plant growth stages. The previously reported *Phg-1, Phg-2*, *Phg-4*, and *Phg-5* loci were validated, and a new QTL named ALS11.1*^AM^* located at the beginning of chromosome Pv11 was reported (de Almeida et al., 2021). All these studies, based on high-throughput genotyping platforms and GWAS, revealed several resistance genes involved in the ALS response. Molecular markers cosegregating with these resistance loci and haplotypes represent a powerful tool for the development of superior varieties with improved levels of ALS resistance.

Domestication has narrowed the genetic diversity of common beans and, in recent decades, plant breeding has accelerated this process decreasing their potential to adapt to changing conditions of biotic and abiotic stress. Common bean wild relatives represent a particular source of variability for many genetically important traits and have been identified as a source of resistance to some biotic stresses, such as bruchids (Kornegay et al., 1993; Osborn et al., 2003), white mold (Mkwaila et al., 2011), common bacterial blight (Beaver et al., 2012) and web blight (Beaver et al., 2012). NWA represents the southern limit of the Andean gene pool of bean and is probably an area of domestication (Figure 2; Kwak and Gepts, 2009; Rodriguez et al., 2015). High levels of genetic diversity in Argentinean wild populations have been reported, suggesting that the Andean gene pool has a large genetic base in this region (Menéndez-Sevillano, 2002; Galván et al., 2006, 2010a). A high level of tolerance to *P. griseola* races was observed in wild beans from NWA with the identification of resistance gene analog sequences (Stenglein, 2007; Galván et al., 2010b). Recent studies based on 34 wild bean populations evaluated with three of the most widely distributed races in the main cultivation areas in Argentina was reported by Aparicio (2020). Resistant and tolerant genotypes were observed depending on the pathotype tested. Three wild genotypes resulted resistant to race 63-7, while the other six genotypes were tolerant. This wild germplasm represents new sources of Andean resistance genes and is of great interest to broader the genetic base of bean cultivars.

## Web blight

Common bean web blight (WB), caused by the basidiomycete fungus *R. solani* Kuhn [teleomorph *Thanatephorus cucumeris* (Frank) Donk] is among the most economically important epidemics, given its level of dispersion in bean production areas in the humid tropics causing significant losses in seed quality and yield (Beaver et al., 2021). Web blight is a limiting factor in Argentina (Vizgarra et al., 2012; Spedaletti et al., 2016) and in other regions of Central America and the Caribbean (Gálvez et al., 1989; Godoy-Lutz et al., 2008; Mora-Umaña et al., 2013), Brazil (Alves de Sousa et al., 2014; Boari et al., 2020; Chavarro-Mesa et al., 2020), and Africa (Wortmann et al., 1998; Masangano and Miles, 2004). WB epidemics are favored by rainy weather, high relative humidity (>80%) and high-to-moderate temperature (30–20°C) (Gálvez et al., 1989). The WB fungus has a wide host range and the capacity to survive saprophytically as sclerotia and mycelium in the soil and on plant debris (Cardoso and Luz, 1981), limiting the effectiveness of crop rotation to control the disease. Rain drops are an important source of WB infection splashing soil particles containing mycelium and sclerotia of the pathogen. The basidial stage of the WB pathogen produce basidiospores which are disseminated and produce small circular lesions on the leaves in the canopy. Under humid and warm weather conditions, the lesions expand into irregularly shaped, water soaked lesions and coalesce giving a scalded appearance to infected plants (Figure 1; Godoy-Lutz et al., 1996).

WB pathogen identification resides on assigning *R. solani* isolates to anastomosis groups (AGs) based on the mycelial compatibility between them (Sneh et al., 1991; Carling, 1996). Currently, 15 AGs, with numerous subgroups, have been reported (Liu and Sinclair, 1993; Carling, 1996; Carling et al., 2002; Sharon et al., 2008), of which AG 1, AG 2, and AG 4 have been associated with common bean WB (Galindo et al., 1982; Gálvez et al., 1989; Tu et al., 1996; Godoy-Lutz et al., 2003, 2008; Yang et al., 2007; Dubey et al., 2014). Some of these AGs were further divided into intraspecific groups (ISGs) based on rDNA-ITS sequence analyses, epidemiological differences and cultural characteristics (AG 1-IA, AG 1-IB, AG 1-IE, AG 1-IF, AG 2-2IV, AG 2-2WB; Godoy-Lutz et al., 2003, 2008). Web blight isolates from different regions of Latin America and the Caribbean, where WB is endemic, have been identified by the analysis of rDNA-ITS sequences (Godoy-Lutz et al., 2003, 2008; Spedaletti et al., 2016). AG1 IE and AG-1 IF isolates have been reported as the most common and aggressive within the AG-1 complex, infecting common bean cultivars with moderate levels of resistance (Godoy-Lutz et al., 2008). However, isolates of AG 2-2WB associated with bean WB in Honduras, Costa Rica, Dominican Republic and Ecuador, have been reported (Godoy-Lutz et al., 2003, 2008; Mora-Umaña et al., 2013).

In Argentina, the molecular identification of *R. solani* causing WB in cultivated bean fields has been reported by Spedaletti et al. (2016). In this study 97 isolates recovered from bean plants showing symptoms of WB were identified as *R. solani* AG 2-2WB by means of specific primers and the phylogenetic analysis of rDNA-ITS sequences. Moreover, a great variability in virulence was observed among the isolates in a pathogenicity assay performed in black bean seedlings using colonized wheat grains as source of inoculum. Thirty-two percent of the isolates resulted as highly virulent on the basis of the disease reaction on foliar tissues and no correlation between virulence and geographical origin was detected. Moreover, a few isolates were aggressive on hypocotyls supporting previous observations (Godoy-Lutz et al., 1996; Valentín Torres et al., 2016). Isolates recovered from wild beans (*Phaseolus vulgaris* var. *aborigineus*) growing in the same area have also been identified as *R. solani* AG 2-2WB (Godoy-Lutz et al., 2003, 2008; Spedaletti et al., 2016).

The use of resistant cultivars is an important factor of an integrated management of WB disease. Beaver et al. (2021) recently reviewed the status of breeding for resistance to WB in common bean and although significant progress has been made, common bean cultivars with high levels of resistance to diverse AG groups are still lacking. There are cultivars that in some countries have moderate levels of resistance to WB while in other countries they are more susceptible to the disease (Poltronieri and Ferreira de Oliveira, 1989), emphasizing the fact that local pathogenic WB isolates, characterized by their anastomosis group, should be used in germplasm screening to allow for the identification of sources of genetic resistance (Beaver et al., 2021). Considering this, 23 common bean cultivars inoculated with two highly virulent AG 2-2 isolates collected in northwestern Argentina were evaluated for WB resistance by Spedaletti et al. (2017). Based on the disease incidence (DI) on foliar tissue, the Leales B30 and Leales CR5 cultivars, developed by the Instituto Nacional de Tecnologia Agropecuaria (INTA) from Argentina, were classified as resistant (1 = DI < 3) to both isolates. The identification of resistant varieties using isolates identified in the NWA region represents a significant contribution to breeding programs aimed at achieving elite cultivars with durable WB resistance.

## Rhizoctonia root rot

Root rot (RR) caused by *Rhizoctonia solani* is among the major diseases affecting the common bean in Argentina and other bean growing areas worldwide (Abawi, 1989; Mathew and Gupta, 1996; Naseri and Mousavi, 2015), particularly in low soil fertility regions, with limited crop rotation and intensive seasonal bean production (Miklas et al., 2006). Rhizoctonia RR symptoms include sunken, reddish-brown lesions on seedling roots and stems (Abawi, 1989), resulting in young seedling damping-off (Figure 1; Reddy et al., 1993; Hagedorn, 1994). Yield losses, resulting in upward to 100%, have been reported (Abawi, 1989; Singh and Schwartz, 2010). *R. solani* is a soil-borne pathogen that spreads from plant to plant through the formation of mycelial bridges between roots and infested soil debris. The pathogen survives on seeds, facilitating long-distance and overwintering dispersal (Abawi, 1989; Schwartz et al., 2005).

Root and hypocotyl rot have been reported to be caused by isolates of *R. solani* AG 1, AG 2, AG 4, and AG 5 (Galindo et al., 1982; Abawi, 1989; Tu et al., 1996; Eken and Demirci, 2004; Nerey et al., 2010; Valentín Torres et al., 2016). Moreover, AG 4 has been reported to be the prevalent group associated with root and hypocotyl rot in Argentina and other common bean growing areas worldwide, such as Brazil, Cuba, Iran, Turkey and the Democratic Republic of the Congo (Muyolo et al., 1993; Meinhardt et al., 2002; Nerey et al., 2010; Haratian et al., 2013; Kiliçoǧlu and Özkoç, 2013; Spedaletti et al., 2017). In Argentina, the presence of various *R. solani* AGs in seed and soil samples from bean fields naturally infested with RR has been reported (Spedaletti et al., 2017). Based on the variability in the rDNA-ITS sequence, most of the isolates (92%) were identified as *R. solani* AG 4, including AG 4 HG-I (20%) and AG 4 HG-III (26%). Moreover, great variability in virulence among the isolates was observed in a pathogenicity approach under controlled conditions toward bean seedlings, and four virulence categories were defined according to the disease reaction on root and foliar tissues. Considering that seed and soil-borne inoculum play a significant role in pathogen dispersal in the region, the use of certified seeds free of sclerotia is essential to reducing the incidence of Rhizoctonia RR disease. *R. solani* AG 4 can affect other commercial crops that are grown in rotation with beans, such as maize and tobacco (Mercado Cárdenas et al., 2015). Mercado Cárdenas et al. (2015) identified *R. solani* AG 4 HG-I and AG 4 HG-III isolates obtained from tobacco plants with damping-off and sore shin symptoms in different localities in NWA. This highlights the importance of using non-host crops in rotational systems that may reduce root rot incidence, leading to improved control.

However, the most effective strategy for controlling Rhizoctonia RR is the use of resistant cultivars. Genetic resistance to *R. solani* has been reported to be controlled by major as well as minor genes with additive effects (Zhao et al., 2005; Oladzad et al., 2019). Thus, screening for resistance to this soil-borne pathogen is challenging since environmental factors can greatly affect phenotypic responses. Some studies on the identification of Rhizoctonia RR-resistant germplasm have been conducted in common bean (Muyolo et al., 1993; Peña et al., 2013; Adesemoye et al., 2018; Oladzad et al., 2019). Peña et al. (2013) identified genotypes with partial resistance to *R. solani* by screening 275 bean lines in a greenhouse assay. Conner et al. (2014) reported five partially resistant cultivars among 37 common bean lines from different market classes evaluated under field conditions. Recently, Oladzad et al. (2019) performed a wide-scale resistance screening across the Andean (ADP; *n* = 273) and Middle American (MDP; *n* = 279) diversity panels. These diversity panels consist of modern genotypes commonly used in production fields and have been developed to represent bean genetic diversity within each gene pool, facilitating genetic analyses (Cichy et al., 2015; Moghaddam et al., 2016). The Rhizoctonia RR resistance responses of 28 genotypes of the ADP and 18 of the MDP were similar or higher than that of the VAX 3 line used as a resistant control. These new sources of resistance to Rhizoctonia RR will be useful parents for common bean breeding programs. Moreover, a GWAS was performed to discover genomic regions associated with Rhizoctonia RR resistance using the ADP and MDP (Oladzad et al., 2019). This study provided evidence for the existence of one major QTL on Pv01 identified in the MDP and another major QTL on Pv02 in the ADP. These regions were associated with gene clusters encoding proteins similar to known disease resistance genes (Oladzad et al., 2019). This information will be useful to develop molecular markers to facilitate the introgression of Rhizoctonia RR resistance into elite cultivars.

## Concluding remarks

Nowadays it is challenging to facilitate the improvement of crops with such global importance like the common bean while developing cultivars that meet the nutritional requirements of a constantly growing world population and that can also adapt to biotic and abiotic stresses, in the current conditions of climate change.

In this review we described the major fungal disease problems that affect common bean production with emphasis in Argentina. Significant advances have been made in pathogen identification and characterization supplying information on their variability, population structure and reproductive behavior in the main common bean production areas in the country. Furthermore, the selection of representative local isolates supported germplasm screening in regional common bean breeding programs for the development of cultivars with durable resistance.

Managing fungal diseases is complex, so these studies contribute to sustainable management strategies such as genetic resistant cultivars, chemical and biological control, and cultural practices aimed at minimizing yield losses due to WM, ALS, WB, and Rhizoctonia RR, in the region. This review assembled information about the best resistant sources of WM (line A 195), ALS (TUC550, MAB 333, MAB 336, TUC180, and TUC241) and WB (Leales B30 and Leales CR5) in Argentina, which is relevant considering that the use of genetic resistant cultivars is the most promising management tool with the most negligible environmental impact. Regarding Rhizoctonia RR, further germplasm screening based on the pathogen diversity observed in the region, should be performed for the identification of resistant genotypes. Moreover, wild bean populations growing in NWA represent a valuable source of new resistance genes to broaden the common bean genetic base against these pathogens. All these genotypes are being considerate as candidates to generate a diverse association panel for a GWAS approach, that will accelerate the identification of markers associated to the resistance genes and their use in bean improvement.

## Author contributions

CA, GT, and MG contributed to the conception and design of these work and wrote the first draft of the manuscript. YS, MA, and EM wrote sections of the manuscript. GT edited the figures. GM and PO-B edited and revised the manuscript. MG and GM performed the funding acquisition. All authors contributed to manuscript revision, read, and approved the submitted version.

## References

[B1] Abadio A. Lima S. Santana M. Salomão T. Sartorato A. Mizubuti E. (2012). Genetic diversity analysis of isolates of the fungal bean pathogen *Pseudocercospora griseola* from central and southern Brazil. *Genet. Mol. Res.* 11 1272–1279. 10.4238/2012.May.14.1 22614356

[B2] Aban C. L. Taboada G. M. Casalderrey N. B. Maggio M. E. Chocobar M. O. Spedaletti Y. A. (2020). Screening common bean germplasm for resistance to genetically diverse *Sclerotinia sclerotiorum* isolates from Argentina. *Acta Sci. Agron.* 42:e42786. 10.4025/actasciagron.v42i1.42786

[B3] Abán C. L. Taboada G. Spedaletti Y. Aparicio M. Curti R. N. Casalderrey N. B. (2018). Molecular, morphological and pathogenic diversity of *Sclerotinia sclerotiorum* isolates from common bean (*Phaseolus vulgaris*) fields in Argentina. *Plant Pathol.* 67 1740–1748. 10.1111/ppa.12880

[B4] Abán C. L. Taboada G. Spedaletti Y. Maita E. Galván M. Z. (2021). Population structure of the fungus *Sclerotinia sclerotiorum* in common bean fields of Argentina. *Eur. J. Plant Pathol.* 160 841–853. 10.1007/s10658-021-02288-7

[B5] Abawi G. (1989). “Root rots,” in *Bean production problems in the tropics*, eds Schwartz H. F. Pastor-Corrales M. A. (Cali: CIAT), 105–120.

[B6] Adams P. B. (1979). Ecology of *Sclerotinia* species. *Phytopathology* 69 896–899. 10.1094/Phyto-69-896

[B7] Adesemoye A. O. Orrell T. Kodati S. (2018). Effect of virulence of root rot pathogens and cultivar resistance on disease occurrence in dry beans. *Plant Heal. Prog.* 19 237–241. 10.1094/PHP-06-18-0034-RS

[B8] Aldrich-Wolfe L. Travers S. Nelson B. D. (2015). Genetic variation of *Sclerotinia sclerotiorum* from multiple crops in the North Central United States. *PLoS One* 10:e0139188. 10.1371/journal.pone.0139188 26417989 PMC4587960

[B9] Alves de Sousa S. Coelho de Oliveira T. de Melo Oliveira Gonçalves G. Barcelos Souza (2014). Journal of biotechnology and biodiversity agronomic characteristics and resistance of common bean genotypes of the mela in southern state Tocantins. *J. Biotec. Biodivers.* 5 130–139. 10.20873/jbb.uft.cemaf.v5n2.sousa

[B10] Aparicio M. (2020). *Variabilidad genética de Pseudocercospora griseola y caracterización de resistencia a mancha angular en poblaciones de poroto silvestre. Facultad de Ciencias Naturales.* Salta: Universidad Nacional de Salta, 144.

[B11] Assefa T. Assibi Mahama A. Brown A. V. Cannon E. K. S. Rubyogo J. C. Rao I. M. (2019). A review of breeding objectives, genomic resources, and marker-assisted methods in common bean (*Phaseolus vulgaris* L.). *Mol. Breed.* 39 1–23. 10.1007/s11032-018-0920-0

[B12] Atallah Z. K. Larget B. Chen X. Johnson D. A. (2004). High genetic diversity, phenotypic uniformity, and evidence of outcrossing in *Sclerotinia sclerotiorum* in the Columbia Basin of Washington State. *Phytopathology* 94 737–742. 10.1094/PHYTO.2004.94.7.737 18943906

[B13] Attanayake R. N. Carter P. A. Jiang D. Del Río-Mendoza L. Chen W. (2013). *Sclerotinia sclerotiorum* populations infecting canola from China and the United States are genetically and phenotypically distinct. *Phytopathology* 103 750–761. 10.1094/PHYTO-07-12-0159-R 23464902

[B14] Attanayake R. N. Tennekoon V. Johnson D. A. Porter L. D. del Río-Mendoza L. Jiang D. (2014). Inferring outcrossing in the homothallic fungus *Sclerotinia sclerotiorum* using linkage disequilibrium decay. *Heredity (Edinb).* 113 353–363. 10.1038/hdy.2014.37 24781807 PMC4181068

[B15] Basavaraja T. Pratap A. Dubey V. Gurumurthy S. Gupta S. Singh N. P. (2020). Molecular and conventional breeding strategies for improving biotic stress resistance in common bean. *Accel. Plant Breed.* 3 389–421. 10.1007/978-3-030-47306-8_13

[B16] Beaver J. S. Martínez Figueroa H. Godoy Lutz G. Estévez de Jensen C. Porch T. G. Rosas J. C. (2021). Breeding for resistance and integrated management of web blight in common bean. *Crop Sci.* 62 20–35. 10.1002/csc2.20658

[B17] Beaver J. S. Zapata M. Alameda M. Porch T. G. Rosas J. C. (2012). Registration of PR0401-259 and PR0650-31 dry bean germplasm lines. *J. Plant Regist.* 6 81–84. 10.3198/jpr2011.05.0283crg

[B18] Beebe S. E. Pastor-Corrales M. (1991). “Breeding for disease resistance,” in *Common beans: Research for crop improvement*, eds van Schoonhoven A. Voysest O. (Cali, CO: CIAT), 561–617.

[B19] Begerow D. Nilsson H. Unterseher M. Maier W. (2010). Current state and perspectives of fungal DNA barcoding and rapid identification procedures. *Appl. Microbiol. Biotechnol.* 87 99–108. 10.1007/s00253-010-2585-4 20405123

[B20] Billiard S. López-Villavicencio M. Hood M. E. Giraud T. (2012). Sex, outcrossing and mating types: Unsolved questions in fungi and beyond. *J. Evol. Biol.* 25 1020–1038. 10.1111/j.1420-9101.2012.02495.x 22515640

[B21] Boari A. de J. Quadros A. F. F. de Lima Nechet K. Kauffmann C. M. (2020). *Mela em feijão-vagem.* Belém: Embrapa.

[B22] Boland G. J. Hall R. (1994). Canadian journal of plant pathology index of plant hosts of *Sclerotinia sclerotiorum* index of plant hosts of *Sclerotinia* sclerotiomm. *Can. J. Plant Pathol.* 16 93–108. 10.1080/07060669409500766

[B23] Bolton M. D. Thomma B. P. H. J. Nelson B. D. (2006). *Sclerotinia sclerotiorum* (Lib.) de Bary: Biology and molecular traits of a cosmopolitan pathogen. *Mol. Plant Pathol.* 7 1–16. 10.1111/j.1364-3703.2005.00316.x 20507424

[B24] Broughton W. J. Hern G. Blair M. Beebe S. Gepts P. Vanderleyden J. (2003). Beans (*Phaseolus* spp)–model food legumes. *Plant Soil* 252 55–128. 10.1023/A:1024146710611

[B25] Brustolin R. Reis E. M. Pedron L. (2016). Longevidade de escleródios de *Sclerotinia sclerotiorum* na superfície do solo no campo. *Summa Phytopathol.* 42 172–174. 10.1590/0100-5405/2131

[B26] Cardona Mejía C. Flor Montoya C. Morales F. Pastor-Corrales M. A. (1995). *Problemas de campo en los cultivos de fríjol en el trópico.* Cali: Centro Internacional de Agricultura Tropical (CIAT).

[B27] Cardoso J. E. Luz E. D. M. N. (1981). *Avanços na pesquisa sobre a mela do feijoeiro no estado do acre: Embrapa acre-séries.* Rio Branco: EMBRAPA.

[B28] Carling D. E. (1996). “Grouping in *Rhizoctonia Solani* by hyphal anastomosis,” in *Rhizoctonia species: Taxonomy, molecular biology, ecology, pathology and disease control*, eds Sneh B. Jabaji-Hare S. Neate S. Dijst G. (Dordrecht: Kluwer Academic Publishers), 35–48. 10.1007/978-94-017-2901-7_3

[B29] Carling D. E. Kuninaga S. Brainard K. A. (2002). Hyphal anastomosis reactions, rDNA-internal transcribed spacer sequences, and virulence levels among subsets of *Rhizoctonia solani* anastomosis group-2 (AG-2) and AG-BI. *Phytopathology* 92 43–50. 10.1094/PHYTO.2002.92.1.43 18944138

[B30] Carpenter M. A. Frampton C. Stewart A. (1999). Genetic variation in New Zealand populations of the plant pathogen *Sclerotinia sclerotiorum*. *New Zeal. J. Crop Hortic. Sci.* 27 13–21. 10.1080/01140671.1999.9514075

[B31] Chataika B. Bokosi J. Kwapata M. Chirwa R. Mwale V. Mnyenyembe P. (2010). Performance of parental genotypes and inheritance of Angular Leaf Spot (*Phaeosariopsis griseola*) resistance in the common bean (*Phaseolus vulgaris*). *Afr. J. Biotechnol.* 9 4398–4406.

[B32] Chavarro-Mesa E. Ceresini P. Pereira D. Vicentini S. Silva T. Ramos-Molina L. (2020). A broad diversity survey of *Rhizoctonia* species from the Brazilian Amazon reveals the prevalence of R. Solani AG-1 IA on signal grass and the new record of AG-1 IF on cowpea and soybeans. *Plant Pathol.* 69 455–466. 10.1111/ppa.13142

[B33] Cichy K. A. Porch T. G. Beaver J. S. Cregan P. Fourie D. Glahn R. P. (2015). A *Phaseolus vulgaris* diversity panel for andean bean improvement. *Crop Sci.* 55 2149–2160. 10.2135/cropsci2014.09.0653

[B34] Clarkson J. P. Coventry E. Kitchen J. Carter H. E. Whipps J. M. (2013). Population structure of *Sclerotinia sclerotiorum* in crop and wild hosts in the UK. *Plant Pathol.* 62 309–324. 10.1111/j.1365-3059.2012.02635.x

[B35] Clarkson J. P. Warmington R. J. Walley P. G. Denton-Giles M. Barbetti M. J. Brodal G. (2017). Population structure of *Sclerotinia* subartica and *Sclerotinia sclerotiorum* in England, Scotland and Norway. *Front. Microbiol.* 8:490. 10.3389/fmicb.2017.00490 28421039 PMC5378995

[B36] Cole H. (1966). Angular leaf spot associated with severe defoliation of red kisney beans (*Phaseolus vulgaris* L.). *Plant Dis. Report.* 50:494.

[B37] Conner R. L. Hou A. Balasubramanian P. McLaren D. L. Henriquez M. A. Chang K. F. (2014). Reaction of dry bean cultivars grown in western Canada to root rot inoculation. *Can. J. Plant Sci.* 94 1219–1230. 10.4141/cjps2013-416

[B38] Corrêa R. X. Good-God P. I. V. Oliveira M. L. P. Silvia N. Moreira M. A. De Barros E. G. (2001). Herança da resistência à mancha-angular do feijoeiro e identificação de marcadores moleculares flanqueando o loco de resistência. *Fitopatol. Bras.* 26 27–32. 10.1590/S0100-41582001000100005

[B39] Cortinovis G. Frascarelli G. Di Vittori V. Papa R. (2020). Current state and perspectives in population genomics of the common bean. *Plants* 9:330. 10.3390/plants9030330 32150958 PMC7154925

[B40] Crous P. W. Braun U. Hunter G. C. Wingfield M. J. Verkley G. J. M. Shin H. D. (2013). Phylogenetic lineages in *Pseudocercospora*. *Stud. Mycol.* 75 37–114. 10.3114/sim0005 24014898 PMC3713886

[B41] Crous P. W. Liebenberg M. M. Braun U. Groenewald J. Z. (2006). Re-evaluating the taxonomic status of *Phaeoisariopsis griseola*, the causal agent of angular leaf spot of bean. *Stud. Mycol.* 55 163–173. 10.3114/sim.55.1.163 18490977 PMC2104728

[B42] Cubeta M. A. Cody B. R. Kohli Y. Kohn L. M. (1997). Clonality in *Sclerotinia sclerotiorum* on infected cabbage in Eastern North Carolina. *Phytopathology* 87 1000–1004. 10.1094/PHYTO.1997.87.10.1000 18945032

[B43] Ddamulira G. Mukankusi C. Ochwo-Ssemakula M. Edema R. Sseruwagi P. Gepts P. (2014). Distribution and Variability of *Pseudocercospora griseola* in Uganda. *J. Agric. Sci.* 6 16–29. 10.5539/jas.v6n6p16

[B44] de Almeida C. P. Arruda N. de Carvalho Paulino J. F. de Freitas G. M. Bonfante G. F. J. Bajay M. M. (2021). Genetic diversity of *Pseudocercospora griseola* resistance loci in common beans. *Trop. Plant Pathol.* 46 129–138. 10.1007/s40858-020-00395-0

[B45] de Carvalho G. A. Paula Junior T. J. Alzate-Marin A. L. Nietsche S. Barros G. E. Moreira M. A. (1998). Inheritance of resistance of the Andean bean line AND-277 to race 63-23 of Inheritance of resistance of the Andean bean line AND-277 to race 63-23 of *Phaeoisariopsis griseola* and identification of a RAPD marker linked to the resistance gene. *Fitopatol. Bras.* 23 482–485.

[B46] De Jesus J. W. C. Do Vale F. X. R. Coelho R. R. Hau B. Zambolim L. Costa L. C. (2007). Effects of angular leaf spot and rust on yield loss of *Phaseolus vulgaris*. *Phytopathology* 91 1045–1053. 10.1094/PHYTO.2001.91.11.1045 18943439

[B47] Dubey S. C. Tripathi A. Upadhyay B. K. Deka U. K. (2014). Diversity of *Rhizoctonia solani* associated with pulse crops in different agro-ecological regions of India. *World J. Microbiol. Biotechnol.* 30 1699–1715. 10.1007/s11274-013-1590-z 24399024

[B48] Dunn A. R. Kikkert J. R. Pethybridge S. J. (2017). Genotypic characteristics in populations of *Sclerotinia sclerotiorum* from New York State. *U.S.A. Ann. Appl. Biol.* 170 219–228. 10.1111/aab.12330

[B49] Eken C. Demirci E. (2004). Anastomosis groups and pathogenicity of *Rhizoctonia* solani and binucleate *Rhizoctonia* isolates from bean in Erzurum. *Turkey. J. Plant Pathol.* 86 49–52. 10.1094/PDIS-02-15-0236-RE 30699508

[B50] Ekins M. G. Hayden H. L. Aitken E. A. B. Goulter K. C. (2011). Population structure of *Sclerotinia sclerotiorum* on sunflower in Australia. *Aust. Plant Pathol.* 40 99–108. 10.1007/s13313-010-0018-6

[B51] Ender M. Kelly J. D. (2005). Identification of QTL associated with white mold resistance in common bean. *Crop Sci.* 45 2482–2490. 10.2135/cropsci2005.0064

[B52] Espeche C. M. Vizgarra O. N. Tarulli L. Araya M. Daniel F. Daniel Ploper L. (2018). Campaña de poroto 2018, análisis y resultados de ensayos. *Rep. Agroind. Mejor. Genético Cultiv. Tucuman. Estac. Exp. Agroind. Obispo Colombres* 149 1–5.

[B53] Faleiro F. G. Ragagnin V. A. Schuster I. Corrêa R. X. Good-God P. I. Brommonshenkel S. H. (2003). Mapeamento de genes de resistência do feijoeiro à ferrugem, antracnose e mancha-angular usando marcadores RAPD. *Fitopatol. Bras.* 28 59–66. 10.1590/S0100-41582003000100009

[B54] FAO (2022). *FAO statistical programme of work 2022. FAO Stat. Program. Work 2022.* Available online at: https://www.fao.org/statistics/es/ (accessed June 27, 2022).

[B55] Faraghati M. Abrinbana M. Ghosta Y. (2022). Genetic structure of *Sclerotinia sclerotiorum* populations from sunflower and cabbage in West Azarbaijan province of Iran. *Sci. Rep.* 12 1–11. 10.1038/s41598-022-13350-7 35662267 PMC9166751

[B56] Fritsche-Neto R. De Souza T. L. P. O. Pereira H. S. De Faria L. C. Melo L. C. Novaes E. (2019). Association mapping in common bean revealed regions associated with anthracnose and angular leaf spot resistance. *Sci. Agric.* 76 321–327. 10.1590/1678-992x-2017-0306

[B57] Galindo J. Abawi G. Thurston H. D. (1982). Variability among isolates of *Rhizoctonia solani* associated with snap bean hypocotyls and soils in New York. *Plant Dis.* 66:390. 10.1094/PD-66-390

[B58] Galván M. Z. Lanteri A. A. Menéndez Sevillano M. C. Balatti P. A. (2010a). Molecular characterization of wild populations and landraces of common bean from northwestern Argentina. *Plant Biosyst.* 144 365–372. 10.1080/11263500903503942

[B59] Galván M. Z. Stenglein S. A. Balatti P. A. (2010b). Common Bean germplasm molecular analysis: A biotechnological approach for breeding. *Am. J. Plant Sci. Biotechnol.* 4 60–69.

[B60] Galván M. Z. Menéndez-Sevillano M. C. De Ron A. M. Santalla M. Balatti P. A. (2006). Genetic diversity among wild common beans from northwestern Argentina based on morpho-agronomic and RAPD data. *Genet. Resour. Crop Evol.* 53 891–900. 10.1007/s10722-004-0981-2

[B61] Gálvez G. Mora B. Pastor Corrales M. (1989). “Web bligh,” in *Bean production problems in the tropics*, eds Schwartz H. Pastor Corrales M. (Cali: CIAT).*

[B62] Gepts P. Aragão F. J. L. De Barros E. Blair M. W. Brondani R. Broughton W. (2008). “Genomics of phaseolus beans, A major source of dietary protein and micronutrients in the tropics,” in *Genomics of tropical crop plants*, eds Moore P. H. Ming R. (New York, NY: Springer), 113–143. 10.1007/978-0-387-71219-2_5

[B63] Godoy-Lutz G. Arias J. Steadman J. R. Eskridge K. M. (1996). Role of natural seed infection by the web blight pathogen in common bean seed damage. Seedling emergence, and early disease development. *Plant Dis.* 80 887–890. 10.1094/PD-80-0887

[B64] Godoy-Lutz G. Kuninaga A. S. Steadman A. J. R. Powers A. K. (2008). Phylogenetic analysis of *Rhizoctonia solani* subgroups associated with web blight symptoms on common bean based on ITS-5.8S rDNA. *J. Gen. Plant Pathol.* 74 32–40. 10.1007/s10327-007-0060-6

[B65] Godoy-Lutz G. Steadman J. R. Higgins B. Powers K. (2003). Genetic variation among isolates of the web blight pathogen of common bean based on PCR-RFLP of the ITS-rDNA region. *Plant Dis.* 87 766–771. 10.1094/PDIS.2003.87.7.766 30812884

[B66] Gomes E. V. Breseguello L. Augusto M. Nasser L. C. B. Petrofeza S. (2011). Microsatellite markers reveal genetic variation within *Sclerotinia sclerotiorum* populations in irrigated dry bean crops in Brazil. *J. Phytopathol.* 159 94–99. 10.1111/j.1439-0434.2010.01724.x

[B67] Gonçalves-Vidigal M. C. Cruz A. S. Garcia A. Kami J. Filho P. S. V. Sousa L. L. (2011). Linkage mapping of the Phg-1 and Co-14 genes for resistance to angular leaf spot and anthracnose in the common bean cultivar AND 277. *Theor. Appl. Genet.* 122 893–903. 10.1007/s00122-010-1496-1 21113774 PMC3043234

[B68] Gonçalves-Vidigal M. C. Cruz A. S. Lacanallo G. F. Vidigal Filho P. S. Sousa L. L. Pacheco C. M. N. A. (2013). Co-segregation analysis and mapping of the anthracnose Co-10 and angular leaf spot Phg-ON disease-resistance genes in the common bean cultivar Ouro Negro. *Theor. Appl. Genet.* 126 2245–2255. 10.1007/s00122-013-2131-8 23760652

[B69] Goncalves-Vidigal M. C. Gilio T. A. S. Valentini G. Vaz-Bisneta M. Vidigal Filho P. S. Song Q. (2020). New Andean source of resistance to anthracnose and angular leaf spot: Fine-mapping of disease-resistance genes in California Dark Red Kidney common bean cultivar. *PLoS One* 15:e0235215. 10.1371/journal.pone.0235215 32598372 PMC7323968

[B70] Goretti D. Bitocchi E. Bellucci E. Rodriguez M. Rau D. Gioia T. (2014). Development of single nucleotide polymorphisms in *Phaseolus vulgaris* and related *Phaseolus* spp. *Mol. Breed.* 33 531–544. 10.1007/s11032-013-9970-5

[B71] Gujaria-Verma N. Ramsay L. Sharpe A. G. Sanderson L.-A. Debouck D. G. Tar’ B. (2016). Gene-based SNP discovery in tepary bean (*Phaseolus acutifolius*) and common bean (*P. vulgaris*) for diversity analysis and comparative mapping. *BCM Genomics* 17:239. 10.1186/s12864-016-2499-3 26979462 PMC4793507

[B72] Guzmán P. Gepts P. Temple S. Mkandawire A. B. C. Gilbertson R. L. (1999). Detection and differentiation of *Phaeoisariopsis griseola* Isolates with the polymerase chain reaction and group-specific primers. *Plant Dis.* 83 37–42. 10.1094/PDIS.1999.83.1.37 30845436

[B73] Guzmán P. Gilbertson R. Nodar I. R. Johnson W. Temple S. Mandala D. (1995). Characterization of variability in the fungus *Phaeoisariopsis griseola* suggest coevolution with the common bean (*Phaseolus vulgaris*). *Phytopathology* 85 600–607. 10.1094/Phyto-85-600

[B74] Hagedorn D. J. (1994). “Rhizoctonia root rot,” in *Compendium of bean diseases*, ed. Hall R. (St Paul: APS Press), 9–13.

[B75] Hagedorn D. J. Wade E. K. (1974). Bean rust and angular leaf spot in Wisconsin. *Plant Dis. Rep.* 58 330–332.

[B76] Hambleton S. Walker C. Kohn L. M. (2002). Previously known from other crops predominate in 1999-2000 samples from Ontario and Quebec soybean. *Can. J. Plant Pathol.* 24 309–315. 10.1080/07060660209507014

[B77] Haratian M. Safaie N. Sharifnabi B. Mahmudi S. B. Ariana A. (2013). Genetic structure of populations of *Rhizoctonia solani* AG-4 from five provinces in Iran. *Plant Pathol.* 62 649–656. 10.1111/j.1365-3059.2012.02665.x

[B78] Hemmati R. Javan-Nikkhah M. Linde C. C. (2009). Population genetic structure of *Sclerotinia sclerotiorum* on canola in Iran. *Eur. J. Plant Pathol.* 125 617–628. 10.1007/s10658-009-9510-7

[B79] Hyten D. L. Song Q. Fickus E. W. Quigley C. V. Lim J.-S. Choi I.-Y. (2010). High-throughput SNP discovery and assay development in common bean. *BCM Genomics* 11:475. 10.1186/1471-2164-11-475 20712881 PMC3091671

[B80] Kamvar Z. N. Amaradasa B. S. Jhala R. Steadman J. Everhart S. E. (2017). Population structure and phenotypic variation of *Sclerotinia sclerotiorum* from dry bean in the United States. *PeerJ.* 5:e4152. 10.7717/peerj.4152 29230376 PMC5723432

[B81] Kamvar Z. N. Everhart S. E. (2019). Something in the agar does not compute: On the discriminatory power of mycelial compatibility in *Sclerotinia sclerotiorum*. *Trop. Plant Pathol.* 44 32–40. 10.1007/s40858-018-0263-8

[B82] Keller B. Manzanares C. Jara C. Lobaton J. D. Studer B. Raatz B. (2015). Fine-mapping of a major QTL controlling angular leaf spot resistance in common bean (*Phaseolus vulgaris* L.). *Theor. Appl. Genet.* 128 813–826. 10.1007/s00122-015-2472-6 25740562 PMC4544502

[B83] Kiliçoǧlu M. Ç Özkoç I. (2013). Phylogenetic analysis of *Rhizoctonia solani* AG-4 isolates from common beans in Black Sea coastal region, Turkey, based on ITS-5.8S rDNA. *Turkish J. Biol.* 37 18–24. 10.3906/biy-1202-17 31411186

[B84] Kohli Y. Morrall R. A. A. Anderson J. B. Kohn L. M. (1992). Local and Trans-Canadian clonal distribution of *Sclerotinia sclerotiorumon* canola. *Phytopathology* 82 875–880. 10.1094/Phyto-82-875

[B85] Kornegay J. Cardona C. Posso C. E. (1993). Inheritance of resistance to Mexican bean weevil in common bean, determined by bioassay and biochemical tests. *Crop Sci.* 33 589–594.

[B86] Kwak M. Gepts P. (2009). Structure of genetic diversity in the two major gene pools of common bean (*Phaseolus vulgaris* L. Fabaceae). *Theor. Appl. Genet.* 118 979–992. 10.1007/s00122-008-0955-4 19130029

[B87] Lehner M. S. Mizubuti E. S. G. (2017). Are *Sclerotinia sclerotiorum* populations from the tropics more variable than those from subtropical and temperate zones? *Trop. Plant Pathol.* 42 61–69. 10.1007/s40858-016-0125-1

[B88] Lehner M. S. De Paula Júnior T. J. Del Ponte E. M. Mizubuti E. S. G. Pethybridge S. J. (2017). Independently founded populations of *Sclerotinia sclerotiorum* from a tropical and a temperate region have similar genetic structure. *PLoS One* 12:e0173915. 10.1371/journal.pone.0173915 28296968 PMC5352009

[B89] Lehner M. S. Paula Júnior T. J. Hora Júnior B. T. Teixeira H. Vieira R. F. Carneiro J. E. S. (2015). Low genetic variability in *Sclerotinia sclerotiorum* populations from common bean fields in Minas Gerais state. Brazil, at regional, local and micro-scales. *Plant Pathol.* 64 921–931. 10.1111/ppa.12322

[B90] Lehner M. Silva R. A. Paula Júnior T. J. Carneiro J. E. S. Mizubuti E. S. G. (2019). The population of *Sclerotinia sclerotiorum* affecting common bean in Brazil is structured by mycelial compatibility groups. *Trop. Plant Pathol.* 44 41–52. 10.1007/s40858-018-0270-9 33934631

[B91] Liu Z. L. Sinclair J. B. (1993). Differentiation of intraspecific groups within anastomosis group 1 of *Rhizoctonia solani* using ribosomal DNA internal transcribed spacer and isozyme comparisons. *Can. J. Plant Pathol.* 15 272–280. 10.1080/07060669309501923

[B92] Mahuku G. S. Henríquez A. Munõz J. Buruchara R. A. (2002a). Molecular markers dispute the existence of the Afro-Andean group of the bean angular leaf spot pathogen. *Phaeoisariopsis griseola*. *Phytopathology* 92 580–589. 10.1094/PHYTO.2002.92.6.580 18944253

[B93] Mahuku G. S. Jara C. Cuasquer J. B. Castellanos G. (2002b). Genetic variability within *Phaeoisariopsis griseola* from central America and its implications for resistance breeding of common bean. *Plant Pathol.* 51 594–604. 10.1046/j.1365-3059.2002.00742.x

[B94] Mahuku G. S. Iglesias ÁM. Jara C. (2009). Genetics of angular leaf spot resistance in the Andean common bean accession G5686 and identification of markers linked to the resistance genes. *Euphytica* 167 381–396. 10.1007/s10681-009-9897-4

[B95] Masangano C. M. Miles C. A. (2004). Factors influencing Farmers’ adoption of kalima bean (*Phaseolus vulgaris* L.) variety in Malawi. *J. Sustain. Agric.* 24 117–129. 10.1300/J064v24n02_10

[B96] Mathew K. A. Gupta S. K. (1996). Studies on web blight of French bean caused by *Rhizoctonia solani* and its management. *Indian J. Mycol. Plant Pathol.* 26 171–177.

[B97] Maxwell J. J. Brick M. A. Byrne P. F. Schwartz H. F. Shan X. Ogg J. B. (2007). Quantitative trait loci linked to white mold resistance in common bean. *Crop Sci.* 47 2285–2294. 10.2135/cropsci2007.01.0022

[B98] McDonald B. A. Linde C. (2002). Pathogen population genetics, evolutionary potential, and durable resistance. *Annu. Rev. Phytopathol.* 40 349–379. 10.1146/annurev.phyto.40.120501.101443 12147764

[B99] Meinhardt L. W. Wulff N. A. Bellato C. M. Tsai S. M. (2002). Genetic analyses of *Rhizoctonia solani* isolates from *Phaseolus vulgaris* grown in the Atlantic rainforest region of São Paulo. *Brazil. Fitopatol. Bras.* 27 259–267. 10.1590/S0100-41582002000300004

[B100] Menéndez-Sevillano M. C. (2002). *Estudio y conservación del germoplasma silvestre y primitivo de Phaseolus vulgaris L. en el noroeste de argentina.* A Coruña: Universidad de Santiago de Compostela.

[B101] Mercado Cárdenas G. E. Galván M. Z. Barrera V. A. Rodriguero M. S. Carmona M. A. March G. J. (2015). Molecular identification and pathogenicity of *Rhizoctonia* spp. from tobacco growing areas in northwestern Argentina. *Trop. Plant Pathol.* 40 160–168. 10.1007/s40858-015-0035-7

[B102] Mert-Türk F. Ipek M. Mermer D. Nicholson P. (2007). Microsatellite and morphological markers reveal genetic variation within a population of *Sclerotinia sclerotiorum* from oilseed rape in the Çanakkale Province of Turkey. *J. Phytopathol.* 155 182–187. 10.1111/j.1439-0434.2007.01223.x

[B103] Miklas P. N. Kelly J. D. Beebe S. E. Blair M. W. (2006). Common bean breeding for resistance against biotic and abiotic stresses: From classical to MAS breeding. *Euphytica* 147 105–131. 10.1007/s10681-006-4600-5

[B104] Miklas P. N. Porter L. D. Kelly J. D. Myers J. R. (2013). Characterization of white mold disease avoidance in common bean. *Eur. J. Plant Pathol.* 135 525–543. 10.1007/s10658-012-0153-8

[B105] Milgroom M. G. (1996). Recombination and the multilocus structure of fungal populations. *Annu. Rev. Phytopathol.* 34 457–477. 10.1146/annurev.phyto.34.1.457 15012552

[B106] Mkwaila W. Terpstra K. A. Ender M. Kelly J. D. (2011). Identification of QTL for agronomic traits and resistance to white mold in wild and landrace germplasm of common bean. *Plant Breed.* 130 665–672. 10.1111/j.1439-0523.2011.01876.x

[B107] Moghaddam S. M. Mamidi S. Osorno J. M. Lee R. Brick M. Kelly J. (2016). Genome-wide association study identifies candidate loci underlying agronomic traits in a middle American diversity panel of common bean. *Plant Genome* 9 1–21. 10.3835/plantgenome2016.02.0012 27902795

[B108] Mora-Umaña F. Barboza N. Alvarado R. Vásquez M. Godoy-Lutz G. Steadman J. R. (2013). Virulence and molecular characterization of Costa Rican isolates of *Rhizoctonia solani* from common bean. *Trop. Plant Pathol.* 38 461–471. 10.1590/S1982-56762013000600001

[B109] Muyolo N. G. Lipps P. E. Schmitthenner A. F. (1993). Reactions of dry bean. Lima bean, and soybean cultivars to *Rhizoctonia* root and hypocotyl rot and web blight. *Plant Dis.* 77:238. 10.1094/PD-77-0234

[B110] Namayanja A. Buruchara R. Mahuku G. Rubaihayo P. Kimani P. Mayanja S. (2006). Inheritance of resistance to angular leaf spot in common bean and validation of the utility of resistance linked markers for marker assisted selection out side the mapping population. *Euphytica* 1513 361–369. 10.1007/s10681-006-9158-8

[B111] Naseri B. Mousavi S. S. (2015). Root rot pathogens in field soil, roots and seeds in relation to common bean (*Phaseolus vulgaris*), disease and seed production. *Int. J. Pest Manage.* 61 60–67. 10.1080/09670874.2014.993001

[B112] Nay M. M. Buendia H. F. Portilla A. E. Studer B. Raatz B. (2018). Angular leaft spot resistence-GWAS of field and greenhouse screenings in Colombia. *Annu. Rep. Bean Improv. Coop.* 61 5–6.

[B113] Nay M. M. Souza T. L. P. O. Raatz B. Mukankusi C. M. Pastor-Corrales M. A. Abreu A. F. B. (2019b). A review of angular leaf spot resistance in common bean. *Crop Sci.* 59 1376–1391. 10.2135/cropsci2018.09.0596 33343018 PMC7680949

[B114] Nay M. M. Mukankusi C. M. Studer B. Raatz B. (2019a). Haplotypes at the Phg-2 locus are determining pathotype-specificity of angular leaf spot resistance in common bean. *Front. Plant Sci.* 10:1126. 10.3389/fpls.2019.01126 31572421 PMC6753878

[B115] Nerey Y. Pannecoucque J. Hernandez H. P. Diaz M. Espinosa R. De Vos S. (2010). *Rhizoctonia* spp. Causing root and hypocotyl rot in *Phaseolus vulgaris* in Cuba. *J. Phytopathol.* 158 236–243. 10.1111/j.1439-0434.2009.01609.x

[B116] Oblessuc P. R. Baroni R. M. Garcia A. A. F. Chioratto A. F. Carbonell S. A. M. Camargo L. E. A. (2012). Mapping of angular leaf spot resistance QTL in common bean (*Phaseolus vulgaris* L.) under different environments. *BMC Genet.* 13:50. 10.1186/1471-2156-13-50 22738188 PMC3464175

[B117] Oblessuc P. R. Cardoso Perseguini J. M. K. Baroni R. M. Chiorato A. F. Carbonell S. A. M. Mondego J. M. C. (2013). Increasing the density of markers around a major QTL controlling resistance to angular leaf spot in common bean. *Theor. Appl. Genet.* 126 2451–2465. 10.1007/s00122-013-2146-1 23832048

[B118] Oblessuc P. R. Matiolli C. C. Chiorato A. F. Camargo L. E. A. Benchimol-Reis L. L. Melotto M. (2015). Common bean reaction to angular leaf spot comprises transcriptional modulation of genes in the ALS10.1 QTL. *Front. Plant Sci.* 6:152. 10.3389/fpls.2015.00152 25815001 PMC4357252

[B119] Oladzad A. Zitnick-Anderson K. Jain S. Simons K. Osorno J. M. McClean P. E. (2019). Genotypes and genomic regions associated with *Rhizoctonia solani* resistance in common bean. *Front. Plant Sci.* 10:956. 10.3389/fpls.2019.00956 31396253 PMC6667560

[B120] Osborn T. C. Hartweck L. M. Harmsen R. H. Vogelzang R. D. Kmiecik K. A. Bliss F. A. (2003). Registration of *Phaseolus vulgaris* genetic stocks with altered seed protein compositions. (Registrations of Genetic Stocks). *Crop Sci.* 43 1570–1572.

[B121] Panullo A. Kamvar Z. N. Miorini J. Steadman J. R. Everhart S. E. (2018). Genetic variation and structure of *Sclerotinia sclerotiorum* populations from soybean in Brazil. *Phytopathol. Trop. Plant Pathol.* 44 53–64. 10.1007/s40858-018-0266-5

[B122] Papa R. Acosta J. Delgado A. Gepts P. (2005). A genome-wide analysis of differentiation between wild and domesticated *Phaseolus vulgaris* from Mesoamerica. *Theor. Appl. Genet.* 111 1147–1158. 10.1007/s00122-005-0045-9 16142467

[B123] Papa R. Gepts P. (2003). Asymmetry of gene flow and differential geographical structure of molecular diversity in wild and domesticated common bean (*Phaseolus vulgaris* L.) from Mesoamerica. *Theor. Appl. Genet.* 116 239–250. 10.1007/s00122-002-1085-z 12582849

[B124] Papa R. Bellucci E. Rossi M. Leonardi S. Rau D. Gepts P. (2007). Tagging the signatures of domestication in common bean (*Phaseolus vulgaris*) by means of pooled DNA samples. *Ann. Bot.* 100 1039–1051. 10.1093/aob/mcm151 17673468 PMC2759209

[B125] Pascual A. Campa A. Pérez-Vega E. Giraldez R. Miklas P. N. Ferreira J. J. (2010). Screening common bean for *Sclerotinia sclerotiorum* resistance to four isolates collected in northern Spain. *Plant Dis.* 94 885–890. 10.1094/PDIS-94-7-0885 30743546

[B126] Pastor-Corrales M. A. Jara C. E. (1995). La evolución de *Phaeoisariopsis griseola* con el frijol común en América Latina. *Fitopatol. Colomb.* 19 15–24.

[B127] Pastor-Corrales M. A. Jara C. Singh S. P. (1998). Pathogenic variation in, sources of, and breeding for resistance to *Phaeoisariopsis griseola* causing angular leaf spot in common bean. *Euphytica* 103 161–171. 10.1023/A:1018350826591

[B128] Peña P. A. Steadman J. R. Eskridge K. M. Urrea C. A. (2013). Identification of sources of resistance to damping-off and early root/hypocotyl damage from *Rhizoctonia solani* in common bean (*Phaseolus vulgaris* L.). *Crop Prot.* 54 92–99. 10.1016/j.cropro.2013.04.014

[B129] Pérez-Vega E. Pascual A. Campa A. Giraldez R. Miklas P. N. Ferreira J. J. (2012). Mapping quantitative trait loci conferring partial physiological resistance to white mold in the common bean RIL population Xana × Cornell 49242. *Mol. Breed.* 29 31–41. 10.1007/s11032-010-9522-1

[B130] Perseguini J. M. K. C. Oblessuc P. R. Rosa J. R. B. F. Gomes K. A. Chiorato A. F. Carbonell S. A. M. (2016). Genome-wide association studies of anthracnose and angular leaf spot resistance in common bean (*Phaseolus vulgaris* L.). *PLoS One* 11:e0150506. 10.1371/journal.pone.0150506 26930078 PMC4773255

[B131] Ploper D. L. González V. Díaz C. G. Vizgarra O. N. (2016). “Enfermedades del poroto causadas por hongos, bacterias y agentes no infecciosos,” in *Manual técnico del cultivo de poroto para el noroeste argentino*, eds In O. Vizgarra, Espech C. Y. Ploper L. D. (Las Lajitas: EEAOC Press, C1), 109–135.

[B132] Ploper L. D. González V. Díaz C. G. Vizgarra O. N. (2002). Manejo de la mancha angular del poroto. *Av. Agroind.* 23 5–9.

[B133] Poltronieri L. S. Ferreira de Oliveira A. F. (1989). *Web blight of bean: Alternatives for control.* Belém: Embrapa.

[B134] Purdy L. H. (1979). *Sclerotinia sclerotiorum* : History, diseases and symptomatology, host range, geographic distribution, and impact. *Phytopathology* 69:875. 10.1094/Phyto-69-875

[B135] Raatz B. Mukankusi C. Lobaton J. Male A. Chisale V. Amsalu B. (2019). Analyses of African common bean (*Phaseolus vulgaris* L.) germplasm using a SNP fingerprinting platform: Diversity, quality control and molecular breeding. *Genet. Resour. Crop Evol.* 66 707–722. 10.1007/s10722-019-00746-0 30956400 PMC6424151

[B136] Reddy M. S. Hynes R. K. Lazarovits G. (1993). Relationship between in vitro growth inhibition of pathogens and suppression of preemergence damping-off and postemergence root rot of white bean seedlings in the greenhouse by bacteria. *Can. J. Microbiol* 40 113–119. 10.1139/m94-018

[B137] Rezaee Danesh Y. Demir S. (2020). Using DNA barcoding in fungal taxonomy. *Yuz. Yıl Univ. J. Agric. Sci.* 30 989–997. 10.29133/yyutbd.751901

[B138] Rezene Y. Tesfaye K. Clare M. Gepts P. (2018). Pathotypes characterization and virulence diversity of *Pseudocercospora griseola* the causal agent of angular leaf spot disease collected from major common bean (*Phaseolus vulgaris* L.) growing areas of Ethiopia. *J. Plant Pathol. Microbiol.* 09 1–6. 10.4172/2157-7471.1000445

[B139] Rodriguez M. Rau D. Bitocchi E. Bellucci E. Biagetti E. Carboni A. (2015). Landscape genetics, adaptive diversity, and population structure in *Phaseolus vulgaris*. *New Phytol.* 209 1781–1794. 10.1111/nph.13713 26526745

[B140] Rossi M. Bitocchi E. Bellucci E. Nanni L. Rau D. Attene G. (2009). Linkage disequilibrium and population structure in wild and domesticated populations of *Phaseolus vulgaris* L. *Evol. Appl.* 2 504–522. 10.1111/j.1752-4571.2009.00082.x 25567895 PMC3352449

[B141] Sabaté D. C. Brandan C. P. Petroselli G. Erra-Balsells R. Audisio M. C. (2018). Biocontrol of *Sclerotinia sclerotiorum* (Lib.) de Bary on common bean by native lipopeptide-producer *Bacillus strains*. *Microbiol. Res.* 211 21–30. 10.1016/j.micres.2018.04.003 29705203

[B142] Sartorato A. Nietsche S. Barros E. D. Moreira M. A. (1999). Inheritance of angular leaf spot resistance and RAPD markers linked to disease resistance gene in common beans. *Annu. Report- Bean Improv. Coop.* 42 21–22.

[B143] Schmutz J. McClean P. E. Mamidi S. Wu G. A. Cannon S. B. Grimwood J. (2014). A reference genome for common bean and genome-wide analysis of dual domestications. *Nat. Genet.* 467 707–713. 10.1038/ng.3008 24908249 PMC7048698

[B144] Schoch C. L. Seifert K. A. Huhndorf S. Robert V. Spouge J. L. Levesque C. A. (2012). Nuclear ribosomal internal transcribed spacer (ITS) region as a universal DNA barcode marker for Fungi. *Proc. Natl. Acad. Sci. U. S. A.* 109 6241–6246. 10.1073/pnas.1117018109 22454494 PMC3341068

[B145] Schoch C. Crous P. Groenewald J. Boehm E. Burgess T. de Gruyter J. (2009). A class-wide phylogenetic assessment of Dothideomycetes. *Stud. Mycol.* 64 1–15–S10. 10.3114/sim.2009.64.01 20169021 PMC2816964

[B146] Schwartz H. F. Galvez G. C. (1980). *Problemas de producción del frijol.* Cali: CIAT.

[B147] Schwartz H. F. Pastor Corrales M. A. (1989). *Bean production problems in the tropics.* Cali: CIAT, 725.

[B148] Schwartz H. F. Otto K. Terán H. Lema M. Singh S. P. (2006). Inheritance of white mold resistance in *Phaseolus vulgaris* × *P. coccineus* Crosses. *Plant Dis.* 90 1167–1170. 10.1094/PD-90-1167 30781097

[B149] Schwartz H. F. Steadman J. R. Hall R. Forster R. L. (2005). *Compendium of bean diseases (No. Ed. 2).* Saint Paul, MIN: American Phytopathological Society (APS Press).

[B150] Serrato-Diaz L. M. Navarro-Monserrat E. D. Rosas J. C. Chilagane L. A. Bayman P. Porch G. T. (2020). Phylogeny of *Pseudocercospora griseola* from Puerto Rico, central America and Tanzania confirms the existence of an afro-andean clade. *Eur. J. Plant Pathol.* 157 533–547. 10.1007/s10658-020-02015-8

[B151] Sexton A. C. Howlett B. J. (2004). Microsatellite markers reveal genetic differentiation among populations of *Sclerotinia sclerotiorum* from Australian canola fields. *Curr. Genet.* 46 357–365. 10.1007/s00294-004-0543-3 15549318

[B152] Sexton A. C. Whitten A. R. Howlett B. J. (2006). Population structure of *Sclerotinia sclerotiorum* in an Australian canola field at flowering and stem-infection stages of the disease cycle. *Genome* 49 1408–1415. 10.1139/g06-101 17426756

[B153] Sharon M. Kuninaga S. Hyakumachi M. Naito S. Sneh B. (2008). Classification of *Rhizoctonia* spp. using rDNA-ITS sequence analysis supports the genetic basis of the classical anastomosis grouping. *Mycoscience* 49 93–114. 10.1007/S10267-007-0394-0

[B154] Silva R. A. Ferro C. G. Lehner M. da S. Paula T. J. Mizubuti E. S. G. (2021). The Population of *Sclerotinia sclerotiorum* in Brazil is structured by mycelial compatibility groups. *Plant Dis.* 105 3376–3384. 10.1094/PDIS-01-21-0110-RE 33934631

[B155] Singh S. P. Schwartz H. F. (2010). Breeding common bean for resistance to diseases: A review. *Crop Sci.* 50 2199–2223. 10.2135/cropsci2009.03.0163

[B156] Singh S. P. Schwartz H. F. Viteri D. Terán H. Otto K. (2014). Introgressing white mold resistance from *Phaseolus coccineus* PI 439534 to common pinto bean. *Crop Sci.* 54 1026–1032. 10.2135/cropsci2013.07.0489

[B157] Singh S. P. Terán H. Lema M. Schwartz H. F. Miklas P. N. (2007). Registration of white mold resistant dry bean germplasm line a 195. *J. Plant Regist.* 1 62–63. 10.3198/jpr2006.10.0643crg

[B158] Singh S. P. Terán H. Schwartz H. F. Otto K. Lema M. (2009). Introgressing white mold resistance from *Phaseolus* species of the secondary gene pool into common bean. *Crop Sci.* 49 1629–1637. 10.2135/cropsci2008.08.0508

[B159] Singh S. P. Terán H. Schwartz H. F. Otto K. Debouck D. G. Roca W. (2013). White Mold–Resistant. Interspecific Common Bean Breeding Line VRW 32 Derived from. *J. Plant Regist.* 7:95. 10.3198/jpr2012.02.0131crg

[B160] Smith J. M. Smith N. H. O’Rourke M. Spratt B. G. (1993). How clonal are bacteria? *Proc. Natl. Acad. Sci.* 90 4384–4388. 10.1073/pnas.90.10.4384 8506277 PMC46515

[B161] Smolińska U. Kowalska B. (2018). Biological control of the soil-borne fungal pathogen Sclerotinia sclerotiorum–a review. *J. Plant Pathol.* 100 1–12. 10.1007/s42161-018-0023-0

[B162] Sneh B. Burpee L. Ogoshi A. (1991). *Identification of Rhizoctonia species.* St. PaulMN: APS press.

[B163] Soule M. Porter L. Medina J. Santana G. P. Blair M. W. Miklas P. N. (2011). Comparative QTL map for white mold resistance in common bean, and characterization of partial resistance in dry bean lines VA19 and I9365-31. *Crop Sci.* 51 123–139. 10.2135/cropsci2010.06.0356

[B164] Souza T. P. O. Gonçalves-Vidigal M. C. Raatz B. Mukankusi C. M. Abreu ÂF. B. Melo L. C. (2016). Major loci controlling resistance to the angular leaf spot of common bean. *Annu. Rep. Bean Improv. Coop* 59 49–50.

[B165] Spedaletti Y. Aparicio M. Mercado Cárdenas G. Rodriguero M. Taboada G. Aban C. (2016). Genetic characterization and pathogenicity of *Rhizoctonia solani* associated with common bean web blight in the main bean growing area of Argentina. *J. Phytopathol.* 164 1054–1063. 10.1111/jph.12526

[B166] Spedaletti Y. Mercado Cárdenas G. Taboada G. Aban C. Aparicio M. Rodriguero M. (2017). Molecular identification and pathogenicity of *Rhizoctonia* spp. recovered from seed and soil samples of the main bean growing area of Argentina. *Aust. J. Crop Sci.* 11 952–959. 10.21475/ajcs17.11.08.pne432

[B167] Steadman J. R. Boland G. J. (2005). “White Mold,” in *Compendium of bean diseases*, 2nd Edn, eds Schwartz R. L. F. H. F. Steadman J. R. Hall R. (St. Paul, MN), 44–46.

[B168] Stenglein S. A. (2007). *Mancha angular del poroto : Variabilidad del agente etiológico Phaeoisariopsis griseola y tolerancia de Phaseolus vulgaris var. aborigineus. Facultad de Agronomía.* Salta: Universidad de Buenos Aires.

[B169] Stenglein S. A. Balatti P. A. (2006). Genetic diversity of *Phaeoisariopsis griseola* in Argentina as revealed by pathogenic and molecular markers. *Physiol. Mol. Plant Pathol.* 68 158–167. 10.1016/j.pmpp.2006.10.001

[B170] Teixeira F. F. Bosco Dos Santos J. Antonio M. Ramalho P. De Fátima Barbosa Abreu Â Teixeira Guimarães C. (2005). QTL mapping for angular leaf spot in common bean using microsatellite markers. *Crop Breed. Appl. Biotechnol.* 5 272–278. 10.12702/1984-7033.v05n03a03

[B171] Terán H. Lema M. Schwartz H. F. Duncan R. Gilbeilson R. Singh S. P. (2006). Modified Petzoldt and Dickson scale for white mold rating of common bean. *Annu. Rep. Bean Improv. Coop.* 49 115–116.

[B172] Terpstra K. A. Kelly J. D. (2008). QTL Analysis of white mold resistance in an inbred backcross mapping population derived from a wild Mexican bean. *Annu. Rep. Bean Improv. Coop.* 51 3–4.

[B173] Tibayrenc M. Ayala F. J. (2012). Reproductive clonality of pathogens: A perspective on pathogenic viruses, bacteria, fungi, and parasitic protozoa. *Proc. Natl. Acad. Sci. U.S.A.* 109 E3305–E3313. 10.1073/pnas.1212452109 22949662 PMC3511763

[B174] Tobar Piñón M. G. Mafi Moghaddam S. Lee R. K. Mérida J. C. V. DeYoung D. J. Reyes B. A. (2021). Genetic diversity of guatemalan climbing bean collections. *Genet. Resour. Crop Evol.* 68 639–656. 10.1007/s10722-020-01013-3

[B175] Tock A. J. Fourie D. Walley P. G. Holub E. B. Soler A. Cichy K. A. (2017). Genome-wide linkage and association mapping of halo blight resistance in common bean to race 6 of the globally important bacterial pathogen. *Front. Plant Sci.* 8:1170. 10.3389/fpls.2017.01170 28736566 PMC5500643

[B176] Tu C. Hsieh T. Chang Y. (1996). “Vegetable diseases incited by *Rhizoctonia* sp,” in *Rhizoctonia species: Taxonomy, molecular biology, ecology, pathology and disease control*, eds Sneh B. Jabaji-Hare S. Neate S. Dijst G. (Dordrecht: Kluwer Academic Publishers), 369–377. 10.1007/978-94-017-2901-7_34

[B177] Valentín Torres S. Vargas M. M. Godoy-Lutz G. Porch T. G. Beaver J. S. (2016). Isolates of *Rhizoctonia solani* can produce both web blight and root rot symptoms in common bean (*Phaseolus vulgaris* L.). *Plant Dis.* 100 1351–1357. 10.1094/PDIS-11-15-1270-RE 30686205

[B178] Vasconcellos R. C. C. Oraguzie O. B. Soler A. Arkwazee H. Myers J. R. Ferreira J. J. (2017). Meta-QTL for resistance to white mold in common bean. *PLoS One* 12:e0171685. 10.1371/journal.pone.0171685 28199342 PMC5310892

[B179] Vidigal Filho P. S. Gonçalves-Vidigal M. C. Vaz Bisneta M. Souza V. B. Gilio T. A. S. Calvi A. A. (2020). Genome-wide association study of resistance to anthracnose and angular leaf spot in Brazilian Mesoamerican and Andean common bean cultivars. *Crop Sci.* 60 2931–2950. 10.1002/csc2.20308

[B180] Vieira R. F. Júnior T. J. P. Carneiro J. E. S. Teixeira H. Queiroz F. N. (2012). Management of white mold in type III common bean with plant spacing and fungicide. *Trop. Plant Pathol.* 37 95–101.

[B181] Vieira R. F. Lima R. C. Teixeira P. H. Paula Júnior T. J. Carneiro J. E. S. Possamai F. (2022). Managing white mold on common bean with type III growth habit by integrating partial resistance, plant density and fungicide. *Plant Dis.* 10.1094/PDIS-11-21-2414-RE [Epub ahead of print]. 35224984

[B182] Viteri D. M. Otto K. Terán H. Schwartz H. F. Singh S. P. (2015). Use of four *Sclerotinia sclerotiorum* isolates of different aggressiveness, three inoculations per plant, and delayed multiple evaluations to select common beans with high levels of white mold resistance. *Euphytica* 204 457–472. 10.1007/s10681-015-1366-7

[B183] Vizgarra O. N. Espeche C. M. Mamani S. Velazquez D. Ploper D. (2012). Consideraciones generales de la campaña de poroto 2012 y resultados de los ensayos evaluados en el Noroeste Argentino. *Av. Agroind.* 33 29–34.

[B184] Vizgarra O. N. Espeche M. C. Ploper L. D. (2011). Evaluación de nuevos materiales de poroto negro con resistencia a la mancha angular. *Av. Agroind.* 32 29–31.

[B185] Vizgarra O. N. Mamani Gonzales S. Y. Espeche C. M. Ploper D. (2016). Evaluación de líneas para la obtención de nuevos cultivares de poroto tipo carioca para el noroeste argentino. *Rev. Ind. y Agrícola Tucumán* 93 1–7.

[B186] Vizgarra O. N. Mamaní Gonzáles S. Y. Espeche C. M. Méndez D. E. Daniel Ploper L. (2018). Descripción para el registro de la nueva variedad de poroto tipo navy bean TUC 150 desarrollada por la estación experimental agroindustrial obispo colombres. *Rev. Ind. y Agríc. Tucumán Tomo* 95 43–47.

[B187] Vizgarra O. Ploper L. Aguirre N. Karlen J. J. (1999). El poroto en la campaña 1999. *Av. Agroind.* 20 41–45.

[B188] Vlasova A. Capella-Gutiérrez S. Rendón-Anaya M. Hernández-Oñate M. Minoche A. E. Erb I. (2016). Genome and transcriptome analysis of the Mesoamerican common bean and the role of gene duplications in establishing tissue and temporal specialization of genes. *Genome Biol.* 17 1–18. 10.1186/s13059-016-0883-6 26911872 PMC4766624

[B189] Wagara I. N. Mwang’ombe A. W. Kimenju J. W. Buruchara R. A. Jamnadass R. Majiwa P. A. O. (2004). Genetic diversity of *Phaeoisariopsis griseola* in Kenya as revealed by AFLP and group-specific primers. *J. Phytopathol.* 152 235–242. 10.1111/j.1439-0434.2004.00836.x

[B190] Wagara I. N. Mwang’ombe A. W. Kimenju J. W. Buruchara R. A. Kimani P. M. (2011). Reaction of selected common bean genotypes to physiological races of *Phaeoisariopsis griseola* occurring in Kenya. *Afr. Crop Sci. J.* 19 343–355.

[B191] Wortmann C. S. Alexander K. R. Eledu C. A. Allen D. J. (1998). *Atlas of common bean (*Phaseolus vulgaris* L.) production in Africa.* Cali: Centro Internacional de Agricultura Tropical (CIAT).

[B192] Yang G. H. Chen J. Y. Pu W. Q. (2007). First report of head rot of cabbage and web-blight of snap bean caused by *Rhizoctonia solani* AG 4 HG-I. *Plant Pathol.* 56:351. 10.1111/j.1365-3059.2007.01543.x

[B193] Zhao G. Ablett G. R. Anderson T. R. Rajcan I. Schaafsma A. W. (2005). Inheritance and genetic mapping of resistance to *Rhizoctonia* root and hypocotyl rot in Soybean. *Crop Sci.* 45 1441–1447. 10.2135/cropsci2004.0560

[B194] Zuiderveen G. H. Padder B. A. Kamfwa K. Song Q. Kelly J. D. (2016). Genome-wide association study of anthracnose resistance in andean beans (*Phaseolus vulgaris*). *PLoS One* 11:e0156391. 10.1371/journal.pone.0156391 27270627 PMC4894742

