# Supplementary material for: Characterization of fungal pathogens and germplasm screening for disease resistance in the main production area of the common bean in Argentina
Source: Front Plant Sci. 2022 Sep 7;13:986247. doi: 10.3389/fpls.2022.986247 (PMC9490223; doi:10.3389/fpls.2022.986247)
Supplement: Supplementary file 1 [file Table_1.docx]

**Supplementary Table 1.** Set of common bean differential cultivars used to define *Pseudocercospora griseola* races.

When the pathogenicity reaction results susceptible based on a 1-9 scale proposed by van Schoonhoven and Pastor-Corrales (1987) the binary code of the susceptible differential cultivars is summed to assign the race number to the *P. griseola* isolate.

| Differential Cultivar | Gene Pool | Seed size | Common bean race | Binary code |
| --- | --- | --- | --- | --- |
| Don Timoteo | Andean | Large | Chile | 1 |
| G 11796 | Andean | Large | Peru | 2 |
| Bolon Bayo | Andean | Large | Peru | 4 |
| Montcalm | Andean | Large | Nueva Granada | 8 |
| Amendoin | Andean | Large | Nueva Granada | 16 |
| G5686 | Andean | Large | Nueva Granada | 32 |
| PAN 72 | Mesoamerican | Small | Mesoamerica | 1 |
| G2858 | Mesoamerican | Medium | Durango | 2 |
| Flor De Mayo | Mesoamerican | Small | Jalisco | 4 |
| Mexico 54 | Mesoamerican | Medium | Jalisco | 8 |
| BAT 332 | Mesoamerican | Small | Mesoamerica | 16 |
| Cornell 49-242 | Mesoamerican | Small | Mesoamerica | 32 |
